# Genetic polymorphism and natural selection of the erythrocyte binding antigen 175 region II in *Plasmodium falciparum* populations from Myanmar and Vietnam

**DOI:** 10.1038/s41598-023-47275-6

**Published:** 2023-11-16

**Authors:** Tuấn Cường Võ, Hương Giang Lê, Jung-Mi Kang, Haung Naw, Won Gi Yoo, Moe Kyaw Myint, Huynh Hong Quang, Byoung-Kuk Na

**Affiliations:** 1 Department of Parasitology and Tropical Medicine, and Institute of Health Science, Gyeongsang National University College of Medicine, Jinju, 52727 Republic of Korea; 2 Department of Convergence Medical Science, Gyeongsang National University, Jinju, 52727 Republic of Korea; 3 Department of Medical Research Pyin Oo Lwin Branch, Pyin Oo Lwin, Myanmar; 4 Tropical Diseases Clinical and Treatment Research Department, Institute of Malariology, Parasitology, and Entomology Quy Nhon, Quy Nhon, Vietnam

**Keywords:** Parasite genetics, Malaria

## Abstract

*Plasmodium falciparum* erythrocyte binding antigen 175 (PfEBA-175) plays essential role in erythrocyte invasion by the parasite and is a leading vaccine candidate. However, its genetic diversity in global isolates is a concern in developing an universal vaccine incorporating this protein. This study aimed to investigate genetic polymorphisms and natural selection of *pfeba-175* region II (RII) in Myanmar and Vietnam *P. falciparum* isolates. Vietnam *pfeba-175* RII displayed a low genetic polymorphism, while Myanmar *pfeba-175* RII showed high levels of genetic diversity across the region. Point mutations, deletion, and recombinations were main factors contributing to genetic diversities in *P. falciparum* populations. Global *pfeba-175* RII revealed similar, but not identical, genetic polymorphisms and natural selection profiles. Despite profiles of amino acid substitutions differed among populations, five major amino acid changes (K279E, E403K, K481I, Q584K, and R664) were commonly detected in global *pfeba-175* RII populations. Haplotype network and genetic differentiation analyses of global *pfeba-175* RII populations demonstrated no geographical relationships. Non-neglectable level of genetic diversity was observed in global *pfeba-175* RII populations, emphasizing the need to consider this when designing an effective vaccine based on this protein. This study underscores the importance of the continuous monitoring of genetic diversity of *pfeba-175* RII in the global *P. falciparum* populations.

## Introduction

Despite a recent decline in morbidity and mortality of global malaria due to improved control measures, this disease remains a major public health burden in Africa and Southeast Asia^1^. Among five species of human-infecting *Plasmodium* species, *P. falciparum* is the deadliest species contributing to the majority of malaria deaths. Recent spreading of resistance strains against antimalarial drugs has been a priority concern for effectively controlling and eliminating malaria^2^. Thus, alternative methods, including effective vaccine development, are urgently needed to cope with falciparum malaria.

Erythrocyte invasion by *Plasmodium* parasites is the first step that is crucial for parasite growth and malaria pathogenesis. Initial attachment of merozoite to erythrocyte is mediated by merozoite surface proteins (MSPs). Tight interactions are then formed between ligands of the merozoite and specific surface receptors on the host’s erythrocytes. Two classes of protein families, Duffy binding-like (DBL) and reticulocyte binding-like (RBL) proteins, have been identified to be critical ligands involved in the formation of initial tight interaction^3^. Among them, erythrocyte binding antigen 175 of *P. falciparum* (PfEBA-175) has gained attention as a leading blood phase vaccine candidate because antibodies against this protein can inhibit its binding to host receptor glycophorin A (GPA) and eventually block invasion of merozoites in vitro^4^. PfEBA-175 protein comprises seven separated regions, namely RI to RVII^5^. The RII of this protein consisting of two cysteine-rich domains (F1 and F2) plays an important function by interacting with GPA directly followed by inducing erythrocyte membrane deformability essential for successful merozoite invasion^6,7^. PfEBA-175 RII is a promising target for vaccine development as antibodies against this protein have been detected in individuals with naturally acquired immunity^8^. In addition, it can induce host immune responses^9^. Moreover, antibodies that block the binding of PfEBA-175 RII with GPA can potentially neutralize parasite invasion^10–12^. However, *pfeba-175* RII identified in *P. falciparum* wild isolates is genetically diverse, suggesting that monitoring genetic variations of this gene is necessary to provide helpful information for developing an effective vaccine based on this protein^13–15^.

The Greater Mekong Subregion (GMS) covering Cambodia, China (Yunnan and Guangxi Provinces), Lao People's Democratic Republic (Lao PDR), Myanmar, Thailand, and Vietnam is a malaria hot spot. Malaria burden in this region has decreased continuously in recent years due to constructive efforts of all GMS countries toward malaria elimination^1^. However, malaria elimination in the GMS is still challenging due to various existing and emerging matters such as changing malaria transmission patterns and spreading of antimalarial drug resistance^16,17^. High levels of genetic diversity of major vaccine candidate antigens such as MSPs, apical membrane antigen-1 (AMA-1), and circumsporozoite surface protein (CSP) have been reported in *P. falciparum* population of GMS countries^18–24^, suggesting remarkable genetic heterogeneity of *P. falciparum* population circulating in the region, which hampers the design of effective vaccines. Similar to these vaccine candidate antigens, PfEBA-175 also shows genetic polymorphisms among *P. falciparum* populations^14,15,25^. However, information about the genetic diversity of *pfeba-175* in *P. falciparum* population in the GMS is limited. Thus, this study aimed to analyze genetic diversity and natural selection of *pfeba-175* RII in *P. falciparum* isolates from two GMS countries, Myanmar and Vietnam, to understand the genetic structure and evolutionary aspect of the *pfeba-175* gene in the population.

## Results

### Amplifications of *pfeba-175* RII

Amplification of *pfeba-175* RII in blood samples was successful. Sizes of amplified products were approximately 1.9 kb on agarose gel. No size variation was detected among isolates. A total of 33 and 59 sequences of *pfeba-175* RII were successfully obtained from *P. falciparum* isolates from Myanmar and Vietnam, respectively. These sequences were subjected to further analyses.

### Genetic polymorphisms in Vietnam *pfeba-175* RII

**Figure 1:**
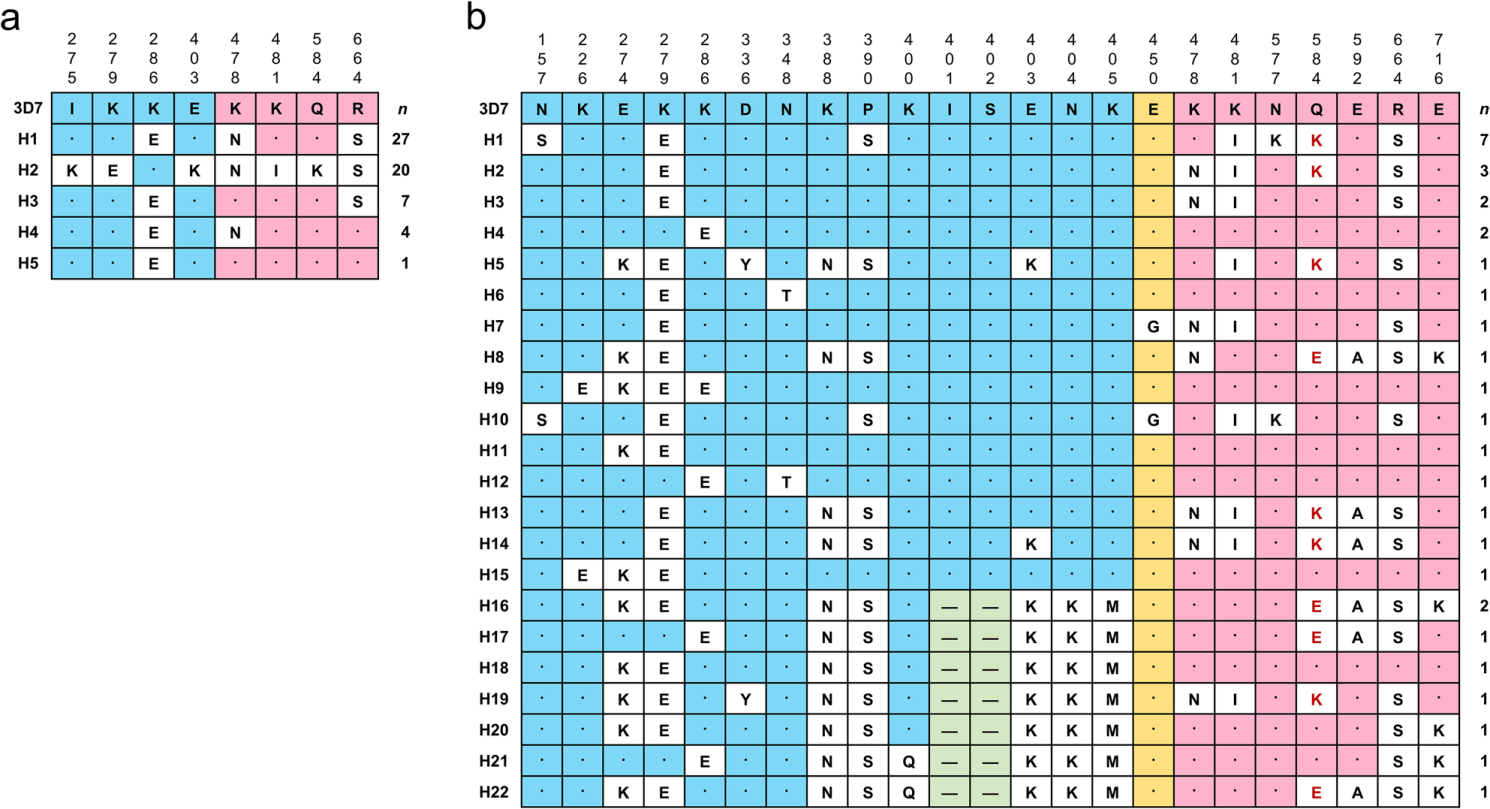
Amino acid changes in Vietnam and Myanmar *pfeba-175* RII. (**a**) Vietnam *pfeba-175* RII. (**b**) Myanmar *pfeba-175* RII. The F1 domain and F2 domain regions were represented with light blue and light red, respectively. The linker region between F1 and F2 domains was denoted with yellow. Dots indicated identical amino acid residues with 3D7 sequence (GenBank ID: XM_001349171.2). The two amino acid deletion (I402 and S402) found in Myanmar *pfeba-175* RII was shown with light green. The positions of amino acid residues were calculated based on the full-length PfEBA-175 (GenBank ID: XM_001349171.2).

Vietnam *pfeba-175* RII covering 616 amino acids was highly conserved among isolates. Compared with the 3D7 reference sequence (GenBank No.: XM_001349171.2), only eight nonsynomous single nucleotide polymorphisms (SNPs) were detected (Supplement File 1: Table S1). These eight SNPs caused dimorphic amino acid substitutions including I275K, K279E, K286E, E403K, K478N, K481I, Q584K, and R664S (Fig. 1a). The first four amino acid changes were detected in the F1 domain, while the remaining four were found in the F2 domain with different proportions. In the F1 domain, the K286E substitution appeared in 39 isolates, accounting for 66.1% of frequency in the total population. The other three amino acid changes, I275K, K279E, and E403K, were observed at lower frequencies (33.9%). On the other hand, K478N and R664S substitutions in the F2 domain showed high frequencies of 86.4% and 91.5%, respectively. Based on these amino acid substitution patterns, Vietnam *pfeba-175* RII sequences were classified into five haplotypes (H1 to H5), none identical to the reference sequence of 3D7. H1 harboring K286E, K478N, and R664S was the most dominant haplotype. H2 was the haplotype showing the highest polymorphic amino acid changes at seven positions.

### Genetic polymorphisms in Myanmar *pfeba-175* RII

Myanmar *pfeba-175* RII sequences exhibited high levels of polymorphisms. A total of 21 nonsynomous SNPs (Supplement File 1: Table S1), resulting in amino acid substitutions at 21 positions (20 dimorphic changes and 1 trimorphic change), were detected (Fig. 1b). Amino acid deletions were also detected at I401 and S402 positions, which were caused by a deletion of six-nucleotides (ATTTCA). Amino acid changes were mainly distributed in the F1 domain, resulting in 13 dimorphic amino acid substitutions (N157S, K226E, E274K, K279E, K286E, D336Y, N348T, K388N, P390S, K400Q, E403K, N404K, and K405M) and two amino acid deletions (I401 and S402). The F2 domain harbored six different dimorphic amino acid substitutions (K478N, K481I, N577K, E592A, R664S, and E716K) and one trimorphic amino acid substitution (Q584E/K). One amino acid change (E450G) was also detected in the linker segment between F1 and F2 domains, albeit its frequency was low (2/33, 6.06%). Based on these amino acid changes, Myanmar *pfeba-175* RII sequences were classified into 22 distinct haplotypes (H1 to H22), different from the reference sequence of 3D7. H1 was the most prevalent haplotype with a frequency of 21.2% (7/33).

### Polymorphism patterns in global *pfeba-175* RII

**Figure 2:**
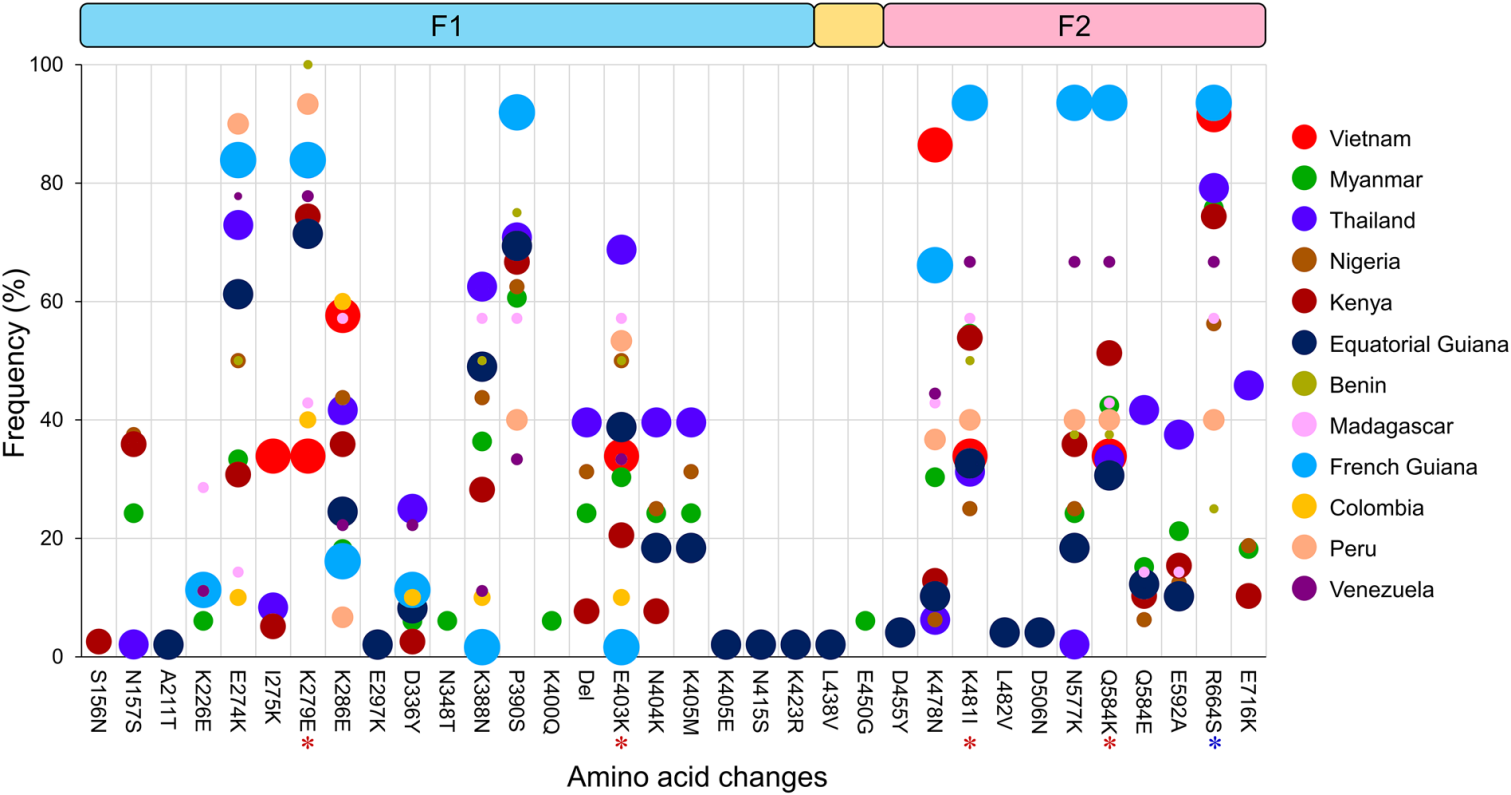
Amino acid changes in the global *pfeba-175* RII. The patterns and frequencies of amino acid substitutions found in the global *pfeba-175* RII were plotted. Each dot with different color represented a different country. The size of the dot denoted the population size. The two amino acids deletion at positions 401 and 402 was presented as the Del. The universal amino acid changes found in all 12 countries were marked as red asterisks. The R664S substitution, which was found in 11 countries, was indicated as a blue asterisk. The regions corresponding to F1, linker, and F2 domains were provided in the figure.

Amino acid polymorphism patterns of global *pfeba-175* RII were comparatively analyzed. The global *pfeba-175* RII population displayed polymorphic characters presenting amino acid changes at 32 positions, including 31 amino acid substitutions and a deletion of two-amino acids in the region (Fig. 2). The F1 domain was more polymorphic than the F2 domain. The F1 domain of global *pfeba-175* RII revealed dimorphic amino acid substitutions at 19 positions (S156N, N157S, A211T, K226E, E274K, I275K, K279E, K286E, E297K, D336Y, N348T, K388N, P390S, K400Q, E403K, N404K, K405M, N415S, and K423R) and one trimorphic amino acid substitution, K405M/E. The F2 domain displayed 9 dimorphic changes (D455Y, K478N, K481I, L482V, D506N, N577K, E592A, R664S, and E716K) and one trimorphic substitution (Q584K/E). These amino acid substitutions were not evenly detected in the global *pfeba-175* RII. Myanmar *pfeba-175* RII showed amino acid changes in the largest number of positions (21 positions). High levels of amino acid changes were also identified in Kenya, Thailand, and Nigeria *pfeba-175* RII at positions of 19, 18, and 17, respectively. Meanwhile, lower amino acid changes were detected in Vietnam, Colombia, and Peru *pfeba-175* RII with 8, 10, and 10 positions, respectively. Only four amino acid changes (K279E, E403K, K481I, and Q584K) were commonly found in all 12 *pfeba-175* RII populations, albeit their frequencies differed by country. High frequencies of R664S were detected in 11 populations except the Equatorial Guiana population, suggesting that it was also a common amino acid substitution found in global *pfeba-175* RII populations. N348T, K400Q, and E450G were uniquely found only in Myanmar *pfeba-175* RII. Deletion of two-amino acids (I401 and S402) observed in the Myanmar population was also observed in Thailand, Nigeria, and Kenya populations with different frequencies. No close geographical relationship of these amino acid substitutions was detected among populations.

### Nucleotide diversity and natural selection of *pfeba-175* RII

**Figure 3:**
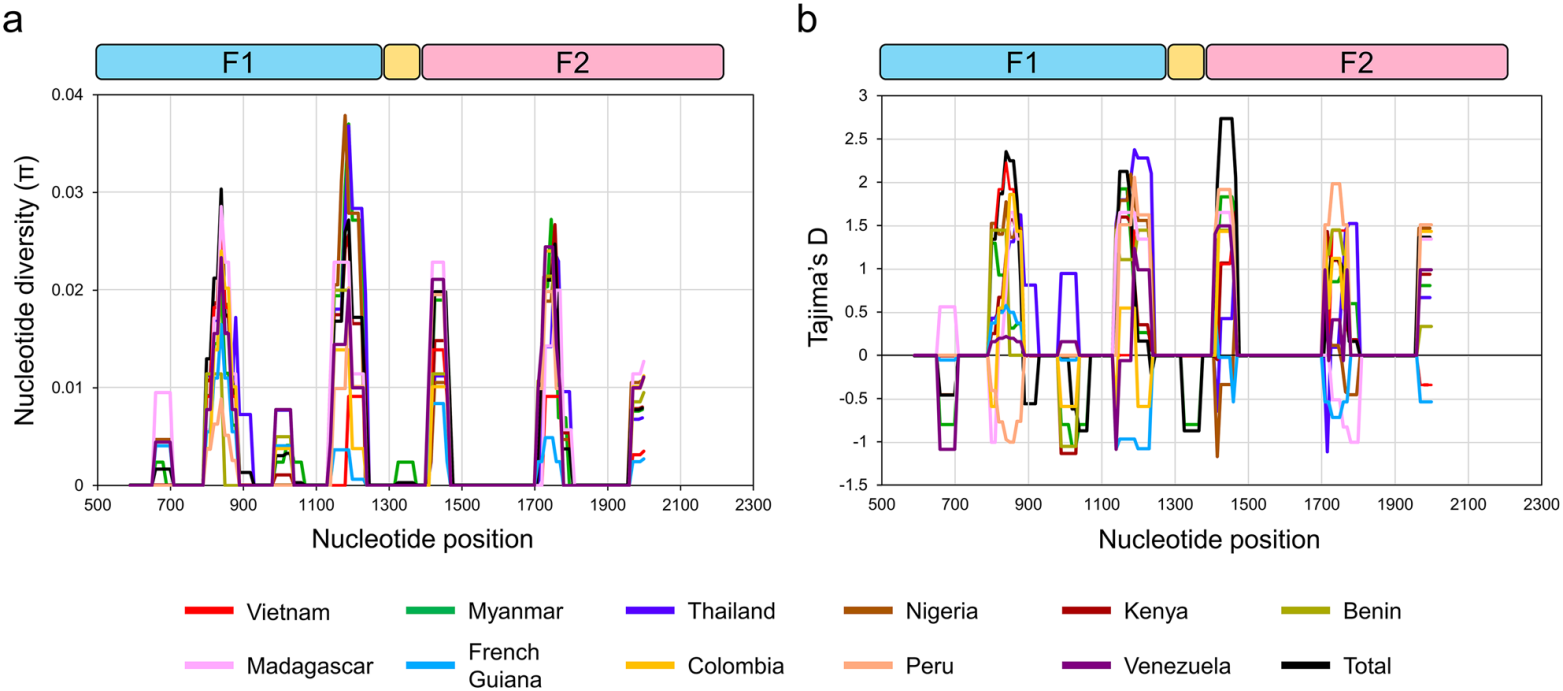
Nucleotide diversity and natural selection of global *pfeba-175* RII. (**a**) Nucleotide diversity. The sliding window plot showed nucleotide diversity (π) values across global *pfeba-175* RII sequences. A window size of 100 bp and a step size of 25 bp was applied. (**b**) Natural selection. The sliding window of Tajima’s D was analyzed for global *pfeba-175* RII. A window size of 100 and a step size of 25 was applied. The patterns of π and Tajima’s D of *pfeba-175* RII from each county were represented with different colors. The regions corresponding to F1, linker, and F2 domains were provided in the figure.

Due to unequal lengths of *pfeba-175* RII sequences from different countries, the same length portion (nucleotide positions from 565 to 2019 of the full-length *pfeba-175* gene) available from 11 countries, including Myanmar and Vietnam, was applied for comparative analysis of genetic diversity and natural selection of global *pfeba-175* RII. Equatorial Guiana sequences were excluded from further analyses because they lacked a large part of the C-terminal region of *pfeba-175* RII. Global *pfeba-175* RII showed similar patterns of genetic diversity and natural selection in global *P. falciparum* populations (Table 1). The highest nucleotide diversity (π) was observed in Myanmar isolates (0.00449 ± 0.00028), while the lowest was found in French Guiana isolates (0.00154 ± 0.00036). The dN–dS values of global *pfeba-175* RII ranged from 0.002 to 0,005, suggesting that weak natural selection influenced global *pfeba-175* RII population. Tajima’s D value was also positive in all isolates except for French Guiana. Fu and Li’s D and Fu and Li’s F values were positive for all analyzed populations. A sliding window plot of π suggested that global *pfeba-175* RII shared highly similar patterns of π across sequences (Fig. 3a). Five significant π peaks were detected, including two peaks (nucleotide positions from 800 to 880 and from 1140 to 1230) in the F1 domain and three peaks (nucleotide positions from 1410 to 1460, from 1710 to 1800, and from 1970 to 2000) in the F2 domain. A sliding window plot of Tajima’s D also showed that global *pfeba-175* RII had a similar pattern of Tajima’s D across the gene, although minor differences were detected among global *pfeba-175* RII (Fig. 3b). High peaks of π matched well with high peaks of Tajima’s D, suggesting a close association between nucleotide diversity and natural selection force across global *pfeba-175* RII. Evidence on natural selection in the global *pfeba-175* RII population was further identified by the McDonald and Kreitman (MD) test (Table 2). The ratio of nonsynonymous to synonymous polymorphic sites within *P. falciparum* (20/2) exhibited a statistically significant increase compared to the ratio observed between *P. falciparum* and *P. vivax* (435/193, *P* < 0.05), as well as between *P. falciparum* and *P. knowlesi* (421/186, *P* < 0.05). These results suggest that *pfeba-175* RII is subject to positive balancing selection. The assertion was also supported by high neutrality scores (4.44 and 4.20, with a significance level of 0.01 < *P* < 0.05). The site-wise selection pressure was also tested by FUBAR test. Positive selection signals (posterior probabilities > 0.9) were predicted in 16 codon sites, including 38, 86, 87, 91, 98, 148, 200, 202, 215, 217, 290, 293, 389, 396, 404, and 476 (Supplement File 2: Table S2). The five common amino acid changes detected in global *pfeba-175* RII were included in these aforementioned sites. Deletion was also one factor contributing to the genetic diversity of global *pfeba-175* RII (Supplement File 3: Table S3). ATTTCA deletion corresponding to I401 and S402 resulted in haplotype diversity, Hd (i), with relatively high values of 0.379, 0.488, and 0.458 in Myanmar, Thailand, and Nigeria *pfeba-175* RII, respectively. Kenya *pfeba-175* RII also exhibited in/del polymorphism with Hd (i) value of 0.146. Recombination also was likely to affect the genetic diversity of global *pfeba-175* RII. The estimated minimum number of recombination events between adjacent polymorphic sites (Rm) in global populations ranged from 0 to 6 (Supplement File 4: Table S4), suggesting that meiotic recombination might contribute to *pfeba-175* RII diversity in some populations such as Myanmar, Thailand, Nigeria, and Kenya. To further analyze the association between SNP positions in *pfepa-175* RII, we tried an linkage disequilibrium (LD) analysis, but no significant association between the loci was found (Supplement File 5: Fig. S1).

**Table 1:** Genetic diversity and natural selection of global *pfeba-175* RII.

Country	*n*	*K*	S	Eta	H	Hd ± SD	π ± SD	dN–dS	Tajima’s D (*P* value)	Fu & Li’s D (*P* value)	Fu & Li’s F (*P* value)
Vietnam	59	3.13	8	8	5	0.668 ± 0.037	0.00215 ± 0.00016	0.003	2.16677 (*P* < 0.05)	1.29151 (*P* > 0.10)	1.85964 (*P* < 0.05)
Myanmar	33	6.51	19	21	22	0.949 ± 0.027	0.00449 ± 0.00028	0.005	0.88890 (*P* > 0.10)	1.34436 (0.05 < *P* < 0.10)	1.40983 (*P* > 0.10)
Thailand	48	6.48	17	18	18	0.900 ± 0.024	0.00447 ± 0.00018	0.004	1.89489 (0.05 < *P* < 0.05)	1.24567 (*P* > 0.10)	1.73796 (*P* < 0.05)
Nigeria	16	6.33	15	16	16	1.000 ± 0.022	0.00437 ± 0.00041	0.005	1.23596 (*P* > 0.10)	0.93001 (*P* > 0.10)	1.16968 (*P* > 0.10)
Kenya	39	5.61	16	17	22	0.903 ± 0.037	0.00387 ± 0.00026	0.005	1.29041 (*P* > 0.10)	1.21530 (*P* > 0.10)	1.46354 (0.05 < *P* < 0.10)
Benin	8	4.46	9	9	7	0.964 ± 0.077	0.00307 ± 0.00040	0.004	1.40165 (*P* > 0.10)	1.11119 (*P* > 0.10)	1.30516 (*P* > 0.10)
Madagascar	7	6.33	12	13	7	1.000 ± 0.076	0.00435 ± 0.00055	0.005	1.06017 (*P* > 0.10)	0.81553 (*P* > 0.10)	0.95645 (*P* > 0.10)
French Guiana	62	2.24	13	13	5	0.365 ± 0.074	0.00154 ± 0.00036	0.002	–0.55758 (*P* > 0.10)	0.43778 (*P* > 0.10)	0.11360 (*P* > 0.10)
Colombia	20	4.48	12	12	3	0.568 ± 0.086	0.00308 ± 0.00053	0.004	1.16929 (*P* > 0.10)	1.46107 (*P* < 0.05)	1.59649 (0.05 < *P* < 0.10)
Peru	30	3.92	10	10	4	0.595 ± 0.057	0.00270 ± 0.00023	0.003	1.75360 (0.05 < *P* < 0.10)	1.40570 (*P* < 0.05)	1.76928 (*P* < 0.05)
Venezuela	9	5.78	14	14	6	0.889 ± 0.091	0.00397 ± 0.00067	0.005	0.58393 (*P* > 0.10)	0.73558 (*P* > 0.10)	0.78221 (*P* > 0.10)
Total	331	6.14	22	24	79	0.931 ± 0.007	0.00424 ± 0.00008	0.005	1.65946 (*P* > 0.10)	1.36639 (*P* > 0.10)	1.79893 (*P* < 0.05)

*n* number of analyzed sequences, *K* average number of nucleotide differences, S number of segregating sites, Eta total number of mutations, H number of haplotypes, Hd haplotype diversity, π observed average pairwise nucleotide diversity, SD standard deviation, dN rate of non–synonymous substitutions, dS rate of synonymous substitutions. *P* < 0.05 indicates statistically significant data.

**Table 2:** McDonald & Kreitman test on the global *pfeba-175* RII with *P. vivax* and *P. knowlesi* erythrocyte binding proteins as outgroup groups.

	Polymorphic changes within *P. falciparum*	Fixed differences between species	
		*Pf* vs *Pv*	*Pf* vs *Pk*
Synonymous substitutions	2	193	186
Nonsynonymous substitutions	20	435	421
Neutrality index		4.44*	4.20*

*Statistically significant Fisher’s exact test *P* value: 0.01 < *P* < 0.05.

### Genetic differentiation between global *pfeba-175* RII

**Figure 4:**
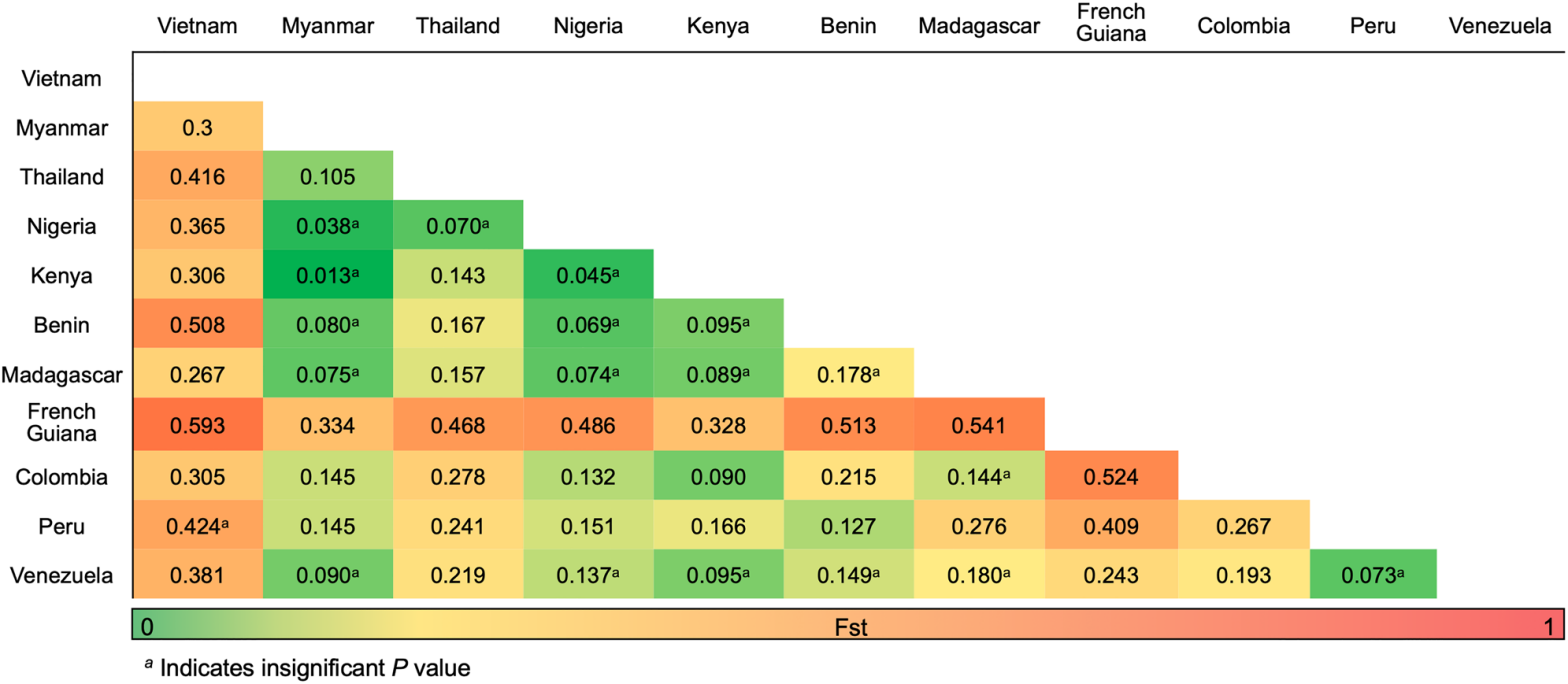
Pair-wise genetic distance (Fst) and gene flow (Nm) between *pfeba-175* RII populations. Pair-wise Fst values are below the diagonal and within population along the diagonal.

To investigate pair-wise genetic differentiation between global *pfeba-175* RII populations, Fst values were calculated (Fig. 4). Fst values among *pfeba-175* RII populations ranged from 0.013 (between Myanmar and Kenya) to 0.593 (between Vietnam and French Guiana). The values between Africa populations except French Guiana were generally low or moderate, suggesting high levels of gene homogeneity within the populations. Interestingly, the values of Vietnam and French Guiana populations against other populations, including populations within the same continent, showed strong genetic differentiations.

### Genetic relationships in the global *pfeba-175* RII

**Figure 5:**
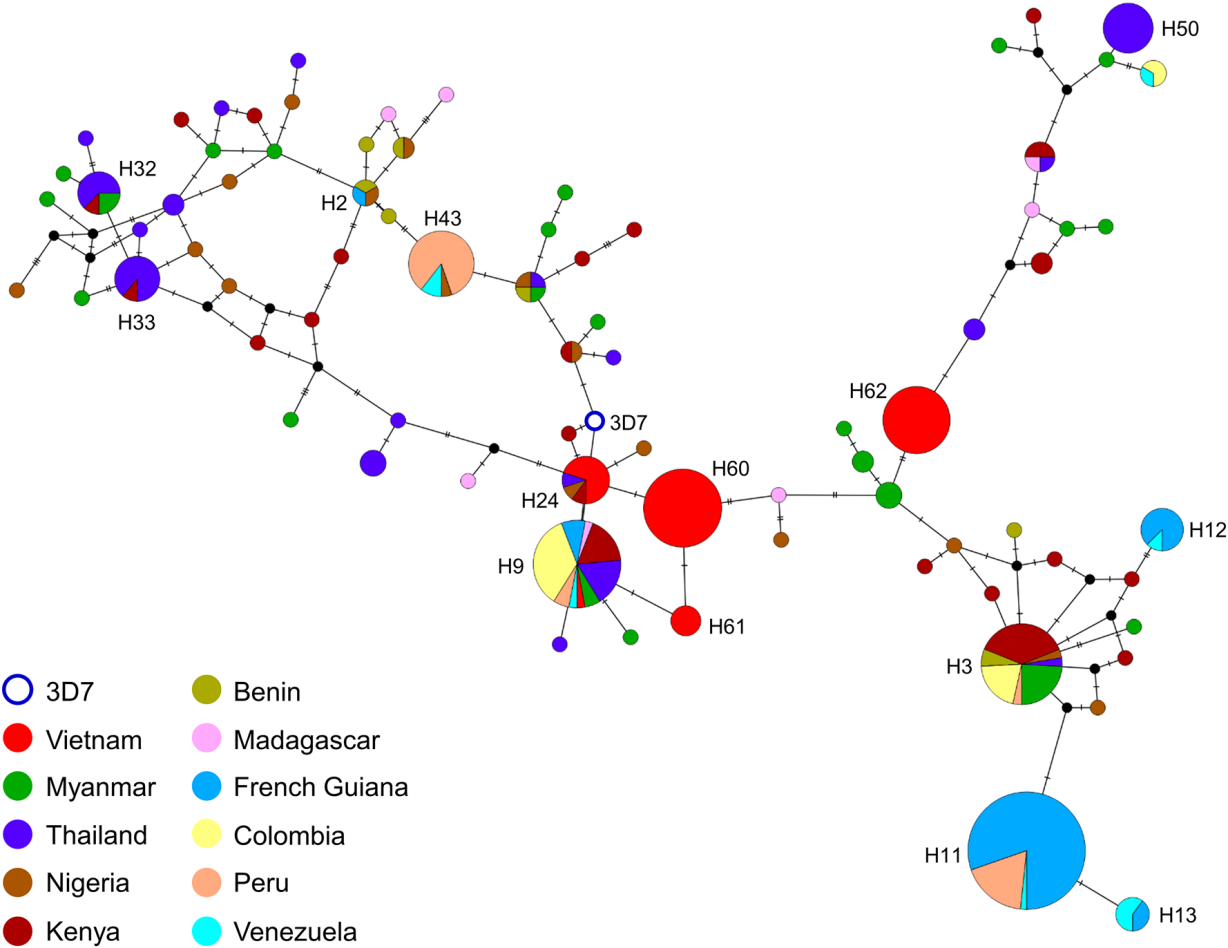
Haplotype network analysis of global *pfeba-175* RII haplotypes. The haplotype network was constructed using the PopART version 1.7^36^ with the Median Joining algorithm^37^. A total of 331 global *pfeba-175* RII sequences and 3D7 reference sequence (XM_001349171.2) were analyzed. The size of each node indicated the frequency of a particular haplotype. The lengths of the lines connecting the nodes were in proportion to the number of base pair substitutions separating the haplotypes. The color of each node indicated each country.

**Figure 6:**
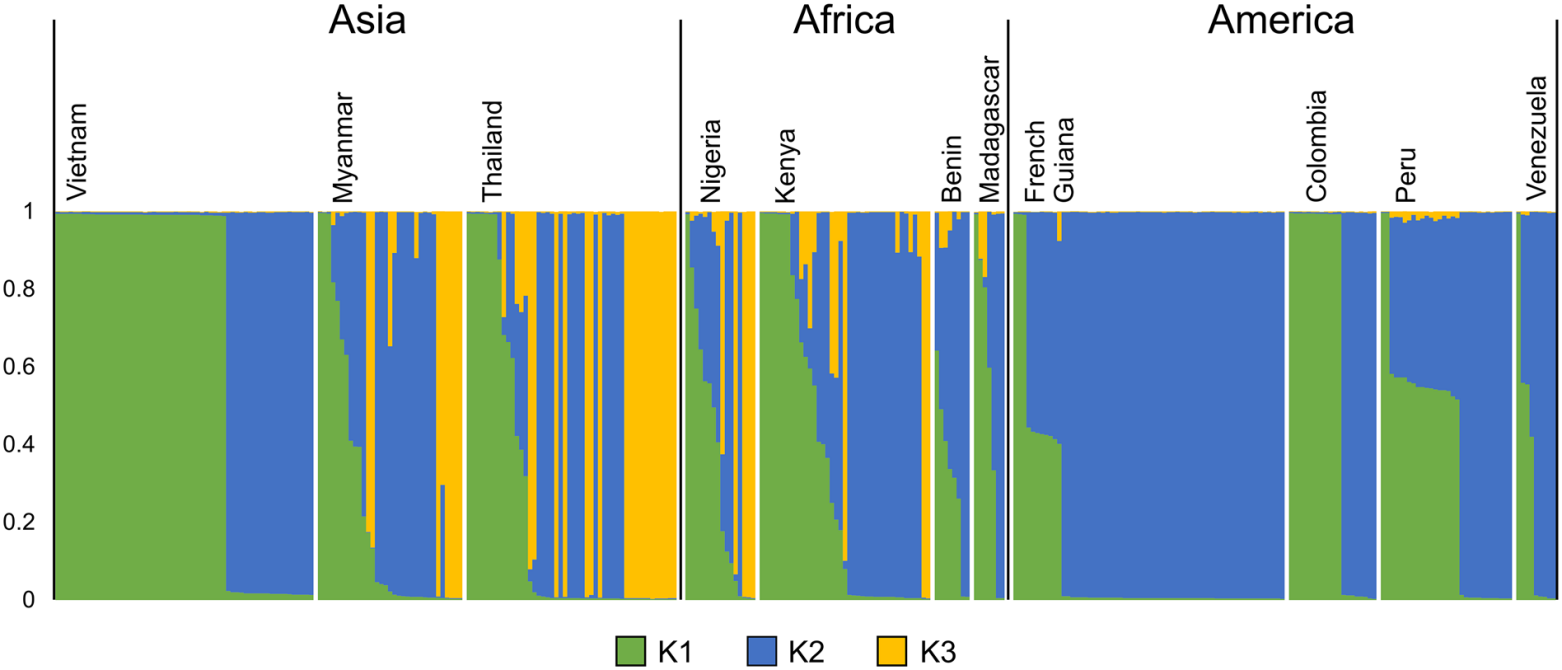
STRUCTURE analysis for global *pfeba-175* RII. Bayesian bar plot showed the structure of *pfeba-175* RII. The *pfeba-175* RII has clustered into three different clusters, K1, K2, and K3. Each cluster was presented by a different color and each bar represented the proportion of each cluster.

Haplotype network analysis revealed that global *pfeba-175* RII (*n* = 331) and the 3D7 reference sequences were clustered into 62 different haplotypes (H1 to H62) connected with a complicated network (Fig. 5). None of global *pfeba-175* RII was identical to reference 3D7. Vietnam *pfeba-175* RII occupied only five distinct haplotypes, H9, H24, H60, H61, and H62, and four of them were closely linked to 3D7. Meanwhile, Myanmar *pfeba-175* RII were divided into 21 haplotypes scattered throughout the network. The *pfeba-175* RII from Africa countries except French Guiana were also broadly dispersed in the network. French Guiana *pfeba-175* RII were grouped into only five separated haplotypes, H2, H9, H11, H12, and H13. The *pfeba-175* RII from America countries also occupied only a few haplotypes. H32 and H33 harboring six nucleotides deletion formed distinct clusters separated from other haplotypes. H9 was the most common haplotype shared by global countries, including Myanmar, Thailand, Vietnam, Kenya, Madagascar, French Guiana, Colombia, Peru, and Venezuela. H3 was also occupied by populations from seven different countries. Cluster analysis using STRUCTURE program was conducted to determine the most accurate number of parasites with similar genotypes in *pfeba-175* RII populations. Data generated from the analysis revealed that 11 global *pfeba-175* RII populations were clustered into three different clusters, K1, K2, and K3 (ΔK = 204.22) (Fig. 6 and Supplemental File 6: Fig. S2). Vietnam *pfeba-175* RII dominantly shared K1 or K2 with an insignificant presence of K3. Similar pattern was also found for *pfeba-175* RII in America populations. On the other hand, Myanmar and Thailand showed an admixture of all three clusters, K1, K2, and K3. Africa population also revealed similar patterns to Myanmar and Thailand populations.

## Discussion

PfEBA-175 RII plays a pivotal role in erythrocyte invasion by merozoite. Thus, it has gained attention as a potential vaccine candidate for falciparum malaria. In this study, we analyzed the genetic diversity of *pfeba-175* RII in Myanmar and Vietnam *P. falciparum* isolates and performed comparative analyses of genetic diversity and evolutionary aspects of *pfeba-175* RII in global populations. Vietnam *pfeba-175* RII was relatively well-conserved with only limited numbers of amino acid changes, while Myanmar *pfeba-175* RII was highly polymorphic harboring amino acid changes at 21 positions. Global *pfeba-175* RII also showed polymorphic characters, of which the F1 domain was more polymorphic than the F2 domain. However, close geographical relationship of these amino acid substitutions was not detected. These amino acid changes were not evenly distributed among global *pfeba-175* RII, but five amino acid changes (K279E, E403K, K481I, Q584K, and R664S) were commonly detected in global *pfeba-175* RII populations with different frequencies by country. Interestingly, a deletion of two amino acids (I401 and S402) was also detected in several populations including Myanmar, Thailand, Nigeria, and Kenya, showing different frequencies. The two residues are likely to be located in the C-terminal region of the F1 domain, which is connected to the linker domain^6^. It is currently unclear whether this deletion could induce significant changes in PfEBA-175 RII structure to alter interaction with GPA. This merits further study.

Global *pfeba-175* RII showed different levels of π values. French Guiana *pfeba-175* RII had the lowest value of 0.00215 ± 0.00016, while Myanmar *pfeba-175* RII showed the highest value of 0.00449 ± 0.00028. However, the slide window plot of π across the gene revealed that global *pfeba-175* RII shared highly similar patterns of π, consistent with a previous report^15^. Positions of five major peaks of π well corresponded to positions of five common amino acid substitutions, suggesting these amino acid changes were major factors contributing to the genetic diversity of global *pfeba-175* RII. The dN–dS values of global *pfeba-175* RII suggested that weak positive natural selection might have influenced global populations, but it was not statistically sifnificant. Values of Tajima's D, Fu and Li’s D, and Fu and Li’s F were positive for most populations, suggesting that balancing selection mainly acted on the gene. Only one exception was the French Guiana population, which showed a negative value of Tajima’s D, implying a weak purifying selection or an expansion in its population size. The balancing positive selection on the global population was also proven via the MD and FUBAR tests. The French Guiana population showed high frequencies of amino acid changes at positions where amino acid substitutions were found, which might have increased genetic homogeneity of the population. Patterns of Tajima’s D in the sliding window plot of the global *pfeba-175* RII were matched well with patterns of π, suggesting these five major common amino acid changes were also major forces affecting the neutrality of global populations. Deletion of two amino acids (I401 and S402) and high recombination events observed in some populations might have also acted as driving forces contributing to the genetic diversity of *pfeba-175* RII. Interestingly, the deletion was only found in Asia and Africa *pfeba-175* RII, not in America population. The overall value of recombination events was also higher for Asia and Africa populations than for America populations. These collectively suggest that the genetic diversity of *pfeba-175* RII is much higher in Asia and Africa *P. falciparum* populations than in America populations.

The critical function of the F2 domain has been reported to be in GPA binding. Amino acid residues in the F2 domain prominently line channels of PfEBA-175 dimer and make the majority of glycan contacts, emphasizing their priority roles in the interaction between PfEBA-175 and GPA^6^. It has also been reported that antibodies against the F2 domain can alter the binding affinity of merozoite to host erythrocytes^10^, indicating the F2 domain could be a major drug or vaccine design target. Recently, it has been reported that recombinant PfEBA-175 RII F2 protein carrying Q584E and E592A favors GPA interaction than recombinant protein with wildtype Q584 and E592^13^. High frequencies of Q584K/E and unneglectable levels of E592A found in global populations suggest that *pfeba-175* RII is evolving to improve its binding ability to GPA. Besides, K481I, N577K, and R664S were also major common amino acid changes detected in the F2 domain in global *pfeba-175* RII. As they are predicted to be located in the interface of GPA binding site^8^ or the dimer interaction site^6^, high levels of polymorphic characters of these residues in global *pfeba-175* RII proposed a hypothesis that *pfeba-175* RII needs these variants to adapt to the host GPA, or the remaining positions receive stronger selection resulting in variants not being retained. This emphasizes the necessity to investigate biological effects of these mutations on structural and functional properties of PfEBA-175. However, considering that limited numbers of sequences from restricted geographical origins were applied in this study, larger-scale of genetic analyses of larger numbers of *P. falciparum* isolates from diverse geographical areas are necessary.

Genetic differentiation between *pfeba-175* RII populations were investigated by estimating Fst values and constructing a haplotype network. Interestingly, Vietnam and French Guiana populations revealed different trends of genetic differentiation from other populations. They showed higher Fst values against other populations, including those in neighboring countries of the same continent, implying that Vietnam and French Guiana populations had strong genetic differentiation from other populations. Haplotype network analysis also supported low genetic relationships of these two populations with other *pfeba-175* RII populations. Most haplotypes of Vietnam and French Guiana populations were clustered into a few unique haplotypes separated from other haplotypes. The reason why these two populations showed such distinct patterns of genetic differentiation and is currently unclear. Such different genetic profile of Vietnam *P. falciparum* population compared to the populations from other neighboring countries in the GMS, including Myanmar and Thailand, has been reported previously^18^. These findings may indicate that the distinctiveness of genetic composition of Vietnam *P. falciparum* was not limited to the *pfeba-175* gene alone but encompasses the other genes in the Vietnam *P. falciparum* population. The geographical features of Vietnam may serve as a contributing component to this phenomenon. The Annamite range (Truong Son) running across the length of Vietnam may act as a natural barrier that separates Vietnam from west Indochina. The height and climatic fluctuations within the range can exert an influence on malaria transmission. Areas at lower altitudes on the windward side may exhibit more favorable conditions for the proliferation of mosquitoes and subsequent malaria transmission, whereas higher-altitude regions on the leeward side may be less conducive to these phenomena. These could affect variations in the prevalence and transmission of malaria on different sides of the range. Furthermore, the geographical characteristics of the range can also exert an influence on patterns of human migration and genetic exchange of malaria parasites across distinct groups. It needs to be clarified in the future.

One more interesting finding of this study was that the Myanmar population showed low genetic differentiation with Africa populations but not with populations from neighboring GMS countries such as Vietnam and Thailand. Considering the great geographical distance between the two countries, it is not easy to deduce the answer. One possible reason for this strange association of Myanmar and Kenya could come from the high sequence similarity of these 2 populations, rather than a biological exchange of individual migrants. Higher genetic diversities of diverse vaccine candidate antigens in the Myanmar *P. falciparum* population compared to populations from the other GMS countries have been reported previously^19–21,26,27^. Further analysis is necessary to understand the high genetic heterogeneity in Myanmar *P. falciparum* population and its evolutionary aspect.

In summary, overall patterns of nucleotide diversity and natural selection in Myanmar and Vietnam *pfeba-175* RII were similar to those in previously reported global populations. However, Myanmar *pfeba-175* RII showed greater nucleotide diversity and larger amino acid changes than the global population. Meanwhile, Vietnam *pfeba-175* RII showed lower genetic diversity and different trend of genetic differentiation compared to populations of neighboring GMS countries, Myanmar and Thailand. Comparative genetic analyses of Lao PDR and Cambodia *pfeba-175* RII populations would contribute to interpreting these genetic differences among GMS countries. Amino acid substitutions, natural selection, deletion, and intragenic recombination might be main forces generating genetic diversity of global *pfeba-175* RII. High or unneglectable level of genetic diversity observed in global *pfeba-175* RII populations, emphasizing the need to consider adaptive selection in *P. falciparum* populations when designing an effective vaccine. The limitation of this study was small and unequal sample size of *P. falciparum* isolates from each country and different sampling periods to generate the sequence data. The global *pfeba-175* RII sequences that were gleaned from the previous studies also span a range of chronological periods. Thus, it is conceivable that the results of this study might not precisely depict the current genetic characteristics of the global *pfeba-175* RII population. These could limit generalizability of the findings in this study. However, results of this study provide an evidence on genetic heterogeneity of global *pfeba-175* RII and the necessity of continuous monitoring the genetic diversity of the gene in the global *P. falciparum* population for developing an effective vaccine based on PfEBA-175.

## Materials and methods

### Blood sample collection and ethics

**Figure 7:**
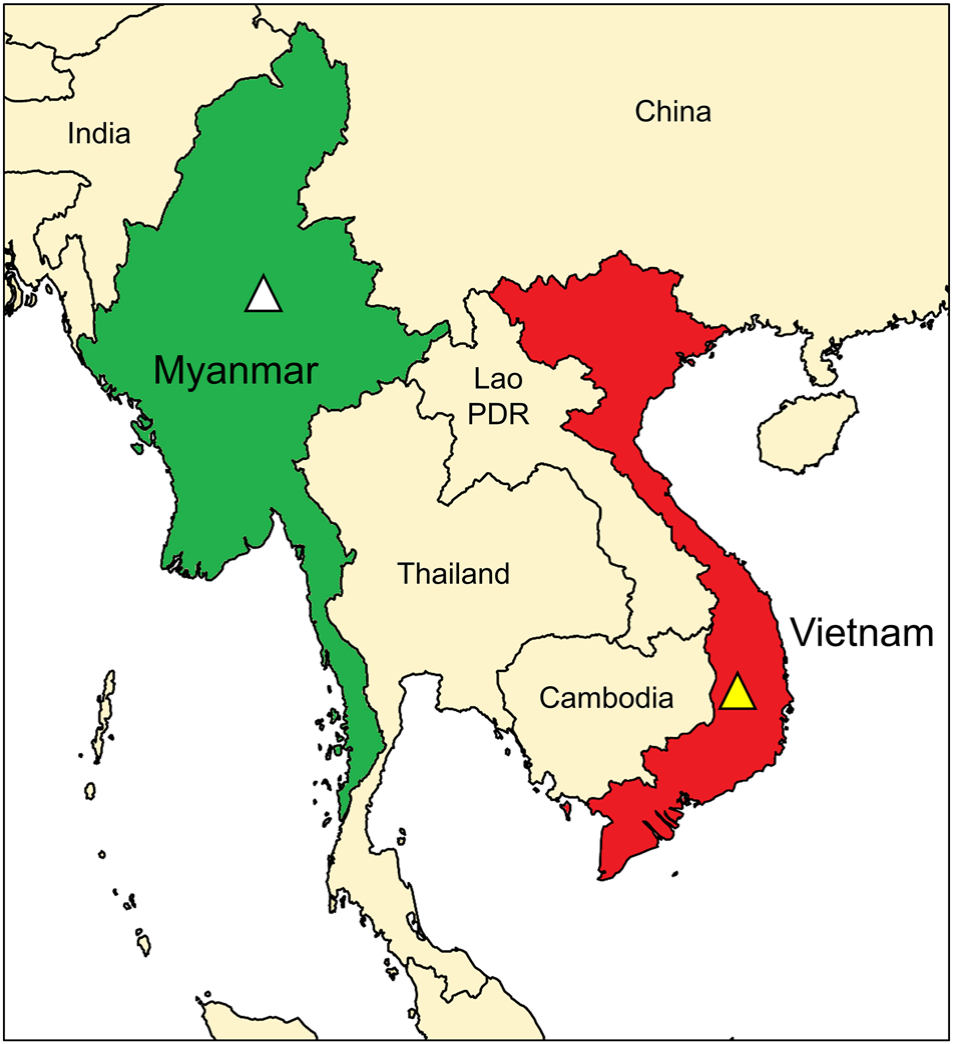
Map of blood collection sites. The blood samples used in this study were collected from malaria patients in Upper Myanmar (white triangle) and Central Highlands in Vietnam (yellow triangle). The map was created with Mapchart (https://www.mapchart.net/).

*P. falciparum*-infected blood samples used in this study were collected from Myanmar and Vietnam (Fig. 7). Myanmar blood samples (*n* = 33) were collected from the patients in Upper Myanmar (Naung Cho, Pyin Oo Lwin, and Tha Beik Kyin) from 2013 to 2015^28^. In Vietnam, blood samples (*n* = 59) were collected from malaria patients residing in four districts in Dak Lak province in the Central Highlands region from 2018 to 2019^29^. The malaria infection was confirmed by species-specific polymerase chain reaction (PCR) targeting the 18S ribosomal RNA (rRNA) gene^29–31^. Monoclonal infection of *P. falciparum* was also confirmed in previous studies^29,31^. The study protocol was approved by the Ministry of Health, Myanmar (97/Ethics 2015) and the Ministry of Health, Institute of Malariology, Parasitology, and Entomology Quy Nhon, Vietnam (No.: 386/VSR-LSDT). All methods were performed in accordance with the relevant guidelines and regulations by the institutions. Informed consent was obtained from all participants and/or their legal guardian(s).

### *pfeba-175* RII amplification and sequencing analysis

DNA was extracted from the dried blood spots using the QIAamp DNA Blood Kit (Qiagen, Hilden, Germany) following the manufacturer’s protocols. Amplification of the *pfeba-175* RII (corresponding amino acid positions from 113 to 760 of PfEBA-175; nucleotide positions from 336 to 2282 of *pfeba-175*) was conducted by using nested PCR. The primers for the first round PCR were 5'-ACAACAATTATCAATCATTTTTATCGA-3' and 5'-TTTCCTCAGGAACTGCTTCTTGCGAA-3'. The thermal cycling parameters for primary PCR were as follows: one cycle of initial denaturation at 95 °C for 5 min, 30 cycles of 94 °C for 1 min, annealing at 58 °C for 2 min, and extension at 72 °C for 2 min, followed by a final extension at 72 °C for 5 min. The nested PCR was performed with primers, 5'-ATCGACAAGTTCATTAATAAAGCAAAA-3' and 5'-GCGAAGTTTGTTCATTATTTCTTATTA-3', at similar amplification conditions, except the annealing temperature of 53 °C. Ex *Taq* DNA polymerase (Takara, Otsu, Japan) with proofreading activity was used in all PCR steps to minimize incorrect nucleotide incorporations during the amplification steps. Each PCR product was separated by electrophoresis on 1.5% agarose gel, and the corresponding amplified product was extracted from the gel and cloned into a T&A cloning vector (Real Biotech Corporation, Banqiao City, Taiwan). Each ligation mixture was transformed into *Escherichia coli* DH5α competent cells, and positive clones with appropriate inserts were selected by colony PCR with the nested PCR primers. The nucleotide sequences of the cloned *pfeba-175* RII were analyzed by the automatic Sanger method with M13 forward and reverse primers. To increase the sequencing accuracy in the middle overlapping areas, two internal primers (5'-GCATGGAGAATTTTTGCTTGAAAG-3' and 5'-TTTTCCTACTAATTTTCTATTGCTC-3') were also applied in the sequencing process. Plasmids from at least two independent clones from each transformation mixture were analyzed to confirm the sequence accuracy. The nucleotide sequences of *pfeba-175* RII obtained in this study have been deposited at GenBank under the accession numbers for Myanmar (OQ680031–OQ680063) and Vietnam (OQ680064–OQ680122).

### Genetic diversity analysis of *pfeba-175* RII

The nucleotide and deduced amino acid sequences of Myanmar and Vietnam *pfeba*-175 were examined using Editseq and Seqman in the DNASTAR package (DNASTAR, Madison, WI, USA). The *pfeba-175* sequence of the 3D7 strain (GenBank ID: XM_001349171.2) was used as the reference. The values of the number of segregating sites (S), haplotypes (H), haplotype diversity (Hd), nucleotide diversity (π), and the average number of pair-wise nucleotide differences within a population (*K*) were calculated with DnaSP ver. 5.10.00^32^. The rates of synonymous (dS) and non-synonymous (dN) substitutions were calculated and compared using the Z-test (*P* < 0.05) with the MEGA6 program^33^ using Nei and Gojobori's method^34^ with the Juke and Cantor (JC) correction of 1,000 bootstrap replications. Based on the acquired values, the dN–dS value was calculated. A positive value for dN–dS implies positive natural selection, whereas a negative value suggests negative or purifying natural selection^35^. Statistical analyses of Tajima’s D, Fu, and Li's D and F were performed using DnaSP ver. 5.10.00^32^ to evaluate the neutral theory of natural selection.

### Genetic diversity of *pfeba-175* RII among global *P. falciparum* isolates

The genetic diversity and natural selection of the global *pfeba-175* RII were analyzed. The sequences of global *pfeba-175* RII available in the public database were used in this study (Supplemental File 7: Table S5). Nucleotide sequence polymorphism analysis and a neutrality test for the global *pfeba-175* RII population were conducted using DnaSP ver. 5.10.00^32^ and MEGA6 program^33^. To better understand the natural selection pressure on the global *pfeba-175* RII, the McDonald & Kreitman test was performed using DnaSP ver. 5.10.00^32^. The erythrocyte binding proteins from *P. vivax* strain Sal I (GenBank ID: DQ156512) and *P. knowlesi* strain H (GenBank ID: XM_039113751.1) were used as out groups. The numbers and parameters of recombination event were also estimated by DnaSP ver. 5.10.00^32^. To investigate site-wise influence of the natural selection in the common region of the global *pfeba-175* RII population, FUBAR analysis was performed using the HyPhy distribution (http://www.hyphy.org/). In addition, to check the associations between the indentified SNPs in the global *pfeba-175* RII population, linkage disequilibrium analysis between these SNP positions was performed and plotted by SRplot (https://www.bioinformatics.com.cn/en). The ARLEQUIN ver 3.5.2.2 program^36^ was used to investigate the molecular variance (AMOVA) to evaluate fixation (Fst). The haplotype network for the global *pfeba-175* RII population was analyzed using the PopART program^37^ with the Median Joining algorithm^38^. The ancestry of the global *pfeba-175* RII was analyzed by STRUCTURE software with a Bayesian method^39^. Three iterations for the number of clusters (*K*) from two to ten were initiated, each with a burning period of 10,000 steps and 20,000 Markov chain Monte Carlo iterations. The best *K* number was determined as previously described^39^ with the aid of Pophelper R Package^40,41^.

### Institutional review board statement

The study was approved by the Ministry of Health, Myanmar (97/Ethics 2015) and the Ministry of Health, Institute of Malariology, Parasitology and Entomology Quy Nhon, Vietnam (No.: 386/VSR-LSDT).

### Supplementary Information

Supplementary Table S1.

Supplementary Table S2.

Supplementary Table S3.

Supplementary Table S4.

Supplementary Figure S1.

Supplementary Figure S2.

Supplementary Table S5.

Supplementary Legends.

## Data Availability

The data supporting the conclusions of this article are provided within the article. The original datasets analyzed in this study are available from the corresponding author upon request. All data generated or analyzed during this study are included in this published article (and its Supplementary Information files). The sequence data obtained in this study are openly available in GenBank of NCBI at https://www.ncbi.nlm.nih.gov/ under the Accession No. OQ680031–OQ680122.
