# Supplementary material for: Genetic polymorphism and natural selection of the erythrocyte binding antigen 175 region II in Plasmodium falciparum populations from Myanmar and Vietnam
Source: Sci Rep. 2023 Nov 16;13:20025. doi: 10.1038/s41598-023-47275-6 (PMC10654615; doi:10.1038/s41598-023-47275-6)
Supplement: Supplementary file 1 — Supplementary Table S1. [file 41598_2023_47275_MOESM1_ESM.pdf]

Supplement File 1: Table S1. Summary of nonsynonymous nucleotide polymorphisms (SNPs) detected in Vietnam and Myanmar *pfeba-175* RII

No	Region	Wild type codon	SNP*	Country		Amino acid change
				Vietnam	Myanmar	
1	F1	AAT	A**G**T		O	N157S
2		AAA	**G**AA		O	K226E
3		GAA	**A**AA		O	E274K
4		ATA	A**A**A	O		I275K
5		AAA	**G**AA	O	O	K279E
6		AAA	**G**AA	O	O	K286E
7		GAT	**T**AT		O	D336Y
8		AAT	A**C**T		O	N348T
9		AAA	AA**T**		O	K388N
10		CCA	**T**CA		O	P390S
11		AAA	**C**AA		O	K400Q
12		GAA	**A**AA	O	O	E403K
13		AAC	AA**A**		O	N404K
14		AAG	A**T**G		O	K405M
15	Linker	GAA	G**G**A		O	E450G
16	F2	AAA	AA**C**	O	O	K478N
17		AAA	A**T**A	O	O	K481I
18		AAT	AA**A**		O	N577K
19		CAA	**A**AA	O	O	Q584K
20		CAA	**G**AA		O	Q584E
21		GAG	G**C**G		O	E592A
22		CGT	**A**GT	O	O	R664S
23		GAA	**A**AA		O	E716K

**Mutated nucleotide position in the codon was marked with a bold underline.*
