# Supplementary material for: Genetic polymorphism and natural selection of the erythrocyte binding antigen 175 region II in Plasmodium falciparum populations from Myanmar and Vietnam
Source: Sci Rep. 2023 Nov 16;13:20025. doi: 10.1038/s41598-023-47275-6 (PMC10654615; doi:10.1038/s41598-023-47275-6)
Supplement: Supplementary file 2 — Supplementary Table S2. [file 41598_2023_47275_MOESM2_ESM.pdf]

Supplement File 2: Table S2. FUBAR test

Site	Partition	α	β	β-α	Prob[α>β]	Prob[α<β]	BayesFactor[α<β]
1	1	1.058	0.518	-0.54	0.558	0.379	0.855
2	1	1.322	0.561	-0.761	0.56	0.378	0.852
3	1	1.262	0.558	-0.704	0.559	0.379	0.856
4	1	2.119	0.514	-1.605	0.585	0.355	0.773
5	1	1.252	0.487	-0.765	0.571	0.366	0.811
6	1	1.047	0.438	-0.608	0.579	0.357	0.778
7	1	1.322	0.561	-0.761	0.56	0.378	0.852
8	1	1.322	0.561	-0.761	0.56	0.378	0.852
9	1	1.441	0.517	-0.923	0.57	0.368	0.818
10	1	0.933	0.459	-0.474	0.567	0.369	0.82
11	1	2.145	0.616	-1.529	0.572	0.367	0.816
12	1	0.933	0.459	-0.474	0.567	0.369	0.82
13	1	1.441	0.517	-0.923	0.57	0.368	0.818
14	1	0.836	0.518	-0.318	0.547	0.388	0.891
15	1	0.619	0.456	-0.163	0.54	0.393	0.908
16	1	2.55	0.486	-2.064	0.598	0.343	0.732
17	1	0.837	0.534	-0.303	0.545	0.391	0.901
18	1	2.118	0.51	-1.607	0.585	0.354	0.77
19	1	2.099	0.465	-1.633	0.596	0.344	0.735
20	1	1.054	0.486	-0.568	0.565	0.372	0.831
21	1	1.322	0.561	-0.761	0.56	0.378	0.852
22	1	1.438	0.505	-0.934	0.572	0.366	0.81
23	1	0.619	0.454	-0.165	0.54	0.392	0.906
24	1	1.251	0.482	-0.769	0.572	0.365	0.807
25	1	1.441	0.517	-0.923	0.57	0.368	0.818
26	1	1.441	0.517	-0.923	0.57	0.368	0.818
27	1	1.438	0.505	-0.934	0.572	0.366	0.81
28	1	1.251	0.482	-0.769	0.572	0.365	0.807
29	1	1.432	0.476	-0.955	0.578	0.359	0.787
30	1	1.252	0.487	-0.765	0.571	0.366	0.811
31	1	0.8	0.81	0.01	0.521	0.415	0.998
32	1	1.252	0.487	-0.765	0.571	0.366	0.811
33	1	1.441	0.517	-0.923	0.57	0.368	0.818
34	1	1.441	0.517	-0.923	0.57	0.368	0.818
35	1	2.119	0.514	-1.605	0.585	0.355	0.773
36	1	2.118	0.51	-1.607	0.585	0.354	0.77
37	1	1.049	0.453	-0.596	0.574	0.362	0.797
38	1	2.212	26.549	24.337	0.015	0.965	38.284
39	1	2.145	0.616	-1.529	0.572	0.367	0.816
40	1	2.119	0.514	-1.605	0.585	0.355	0.773
41	1	1.251	0.482	-0.769	0.572	0.365	0.807
42	1	1.441	0.517	-0.923	0.57	0.368	0.818
43	1	2.144	0.607	-1.537	0.573	0.367	0.813
44	1	2.127	0.538	-1.589	0.581	0.359	0.786
45	1	2.119	0.514	-1.605	0.585	0.355	0.773

46	1	2.118	0.51	-1.607	0.585	0.354	0.77
47	1	0.8	0.81	0.01	0.521	0.415	0.998
48	1	0.837	0.534	-0.303	0.545	0.391	0.901
49	1	2.119	0.514	-1.605	0.585	0.355	0.773
50	1	2.107	0.484	-1.623	0.591	0.349	0.752
51	1	2.144	0.607	-1.537	0.573	0.367	0.813
52	1	1.248	0.711	-0.537	0.546	0.392	0.906
53	1	2.118	0.51	-1.607	0.585	0.354	0.77
54	1	2.145	0.616	-1.529	0.572	0.367	0.816
55	1	0.936	0.495	-0.441	0.557	0.379	0.856
56	1	2.099	0.465	-1.633	0.596	0.344	0.735
57	1	1.252	0.487	-0.765	0.571	0.366	0.811
58	1	1.246	0.459	-0.787	0.579	0.358	0.784
59	1	2.55	0.486	-2.064	0.598	0.343	0.732
60	1	0.936	0.495	-0.441	0.557	0.379	0.856
61	1	2.119	0.514	-1.605	0.585	0.355	0.773
62	1	2.118	0.51	-1.607	0.585	0.354	0.77
63	1	2.55	0.486	-2.064	0.598	0.343	0.732
64	1	2.118	0.51	-1.607	0.585	0.354	0.77
65	1	2.144	0.607	-1.537	0.573	0.367	0.813
66	1	0.936	0.495	-0.441	0.557	0.379	0.856
67	1	1.253	0.493	-0.76	0.57	0.368	0.816
68	1	2.145	0.616	-1.529	0.572	0.367	0.816
69	1	0.949	0.783	-0.166	0.531	0.406	0.961
70	1	1.246	0.461	-0.786	0.578	0.359	0.786
71	1	0.837	0.534	-0.303	0.545	0.391	0.901
72	1	0.932	0.457	-0.476	0.568	0.368	0.818
73	1	1.438	0.505	-0.934	0.572	0.366	0.81
74	1	1.049	0.453	-0.596	0.574	0.362	0.797
75	1	1.441	0.517	-0.923	0.57	0.368	0.818
76	1	1.322	0.561	-0.761	0.56	0.378	0.852
77	1	1.432	0.476	-0.955	0.578	0.359	0.787
78	1	1.438	0.505	-0.934	0.572	0.366	0.81
79	1	1.262	0.558	-0.704	0.559	0.379	0.856
80	1	2.144	0.607	-1.537	0.573	0.367	0.813
81	1	1.441	0.517	-0.923	0.57	0.368	0.818
82	1	0.774	0.489	-0.285	0.549	0.386	0.884
83	1	1.246	0.459	-0.787	0.579	0.358	0.784
84	1	2.099	0.465	-1.633	0.596	0.344	0.735
85	1	0.774	0.489	-0.285	0.549	0.386	0.884
86	1	2.2	41.353	39.153	0.003	0.985	91.554
87	1	1.818	23.632	21.813	0.013	0.968	42.334
88	1	2.107	0.484	-1.623	0.591	0.349	0.752
89	1	1.438	0.505	-0.934	0.572	0.366	0.81
90	1	2.099	0.465	-1.633	0.596	0.344	0.735
91	1	2.177	33.38	31.204	0.008	0.977	58.77
92	1	1.322	0.561	-0.761	0.56	0.378	0.852
93	1	1.441	0.517	-0.923	0.57	0.368	0.818
94	1	2.119	0.514	-1.605	0.585	0.355	0.773

95	1	2.144	0.607	-1.537	0.573	0.367	0.813
96	1	1.078	0.497	-0.581	0.563	0.374	0.838
97	1	1.441	0.517	-0.923	0.57	0.368	0.818
98	1	2.252	49.454	47.202	0	0.991	155.304
99	1	2.628	0.772	-1.856	0.571	0.37	0.826
100	1	2.628	0.772	-1.856	0.571	0.37	0.826
101	1	2.119	0.514	-1.605	0.585	0.355	0.773
102	1	1.438	0.505	-0.934	0.572	0.366	0.81
103	1	2.144	0.607	-1.537	0.573	0.367	0.813
104	1	1.078	0.497	-0.581	0.563	0.374	0.838
105	1	0.836	0.518	-0.318	0.547	0.388	0.891
106	1	1.441	0.517	-0.923	0.57	0.368	0.818
107	1	1.252	0.487	-0.765	0.571	0.366	0.811
108	1	2.628	0.772	-1.856	0.571	0.37	0.826
109	1	1.438	0.505	-0.934	0.572	0.366	0.81
110	1	1.246	0.459	-0.787	0.579	0.358	0.784
111	1	2.55	0.486	-2.064	0.598	0.343	0.732
112	1	12.55	0.896	-11.653	0.874	0.099	0.154
113	1	1.251	0.482	-0.769	0.572	0.365	0.807
114	1	0.836	0.518	-0.318	0.547	0.388	0.891
115	1	2.099	0.465	-1.633	0.596	0.344	0.735
116	1	1.441	0.517	-0.923	0.57	0.368	0.818
117	1	2.119	0.514	-1.605	0.585	0.355	0.773
118	1	2.119	0.514	-1.605	0.585	0.355	0.773
119	1	1.224	0.532	-0.692	0.561	0.376	0.846
120	1	2.119	0.514	-1.605	0.585	0.355	0.773
121	1	2.119	0.514	-1.605	0.585	0.355	0.773
122	1	2.127	0.538	-1.589	0.581	0.359	0.786
123	1	1.441	0.517	-0.923	0.57	0.368	0.818
124	1	2.119	0.514	-1.605	0.585	0.355	0.773
125	1	1.322	0.561	-0.761	0.56	0.378	0.852
126	1	0.618	0.429	-0.189	0.55	0.382	0.867
127	1	1.432	0.476	-0.955	0.578	0.359	0.787
128	1	1.438	0.505	-0.934	0.572	0.366	0.81
129	1	1.438	0.505	-0.934	0.572	0.366	0.81
130	1	1.248	0.711	-0.537	0.546	0.392	0.906
131	1	1.432	0.476	-0.955	0.578	0.359	0.787
132	1	1.322	0.561	-0.761	0.56	0.378	0.852
133	1	1.246	0.461	-0.786	0.578	0.359	0.786
134	1	1.432	0.476	-0.955	0.578	0.359	0.787
135	1	2.628	0.772	-1.856	0.571	0.37	0.826
136	1	1.224	0.532	-0.692	0.561	0.376	0.846
137	1	1.441	0.517	-0.923	0.57	0.368	0.818
138	1	1.438	0.505	-0.934	0.572	0.366	0.81
139	1	2.628	0.772	-1.856	0.571	0.37	0.826
140	1	2.099	0.465	-1.633	0.596	0.344	0.735
141	1	0.936	0.495	-0.441	0.557	0.379	0.856
142	1	1.438	0.505	-0.934	0.572	0.366	0.81
143	1	2.144	0.607	-1.537	0.573	0.367	0.813

144	1	0.8	0.81	0.01	0.521	0.415	0.998
145	1	1.252	0.487	-0.765	0.571	0.366	0.811
146	1	1.438	0.505	-0.934	0.572	0.366	0.81
147	1	1.078	0.497	-0.581	0.563	0.374	0.838
148	1	3.629	21.103	17.475	0.049	0.915	15.079
149	1	2.119	0.514	-1.605	0.585	0.355	0.773
150	1	1.078	0.497	-0.581	0.563	0.374	0.838
151	1	0.949	0.783	-0.166	0.531	0.406	0.961
152	1	1.441	0.517	-0.923	0.57	0.368	0.818
153	1	0.8	0.81	0.01	0.521	0.415	0.998
154	1	0.929	0.431	-0.498	0.577	0.358	0.783
155	1	1.441	0.517	-0.923	0.57	0.368	0.818
156	1	2.107	0.484	-1.623	0.591	0.349	0.752
157	1	1.441	0.517	-0.923	0.57	0.368	0.818
158	1	2.127	0.538	-1.589	0.581	0.359	0.786
159	1	1.441	0.517	-0.923	0.57	0.368	0.818
160	1	3.347	7.536	4.189	0.16	0.791	5.314
161	1	2.119	0.514	-1.605	0.585	0.355	0.773
162	1	0.933	0.459	-0.474	0.567	0.369	0.82
163	1	1.248	0.711	-0.537	0.546	0.392	0.906
164	1	2.145	0.616	-1.529	0.572	0.367	0.816
165	1	1.438	0.505	-0.934	0.572	0.366	0.81
166	1	0.932	0.457	-0.476	0.568	0.368	0.818
167	1	2.127	0.538	-1.589	0.581	0.359	0.786
168	1	0.836	0.518	-0.318	0.547	0.388	0.891
169	1	0.837	0.534	-0.303	0.545	0.391	0.901
170	1	1.438	0.505	-0.934	0.572	0.366	0.81
171	1	2.127	0.538	-1.589	0.581	0.359	0.786
172	1	1.322	0.561	-0.761	0.56	0.378	0.852
173	1	2.118	0.51	-1.607	0.585	0.354	0.77
174	1	0.929	0.431	-0.498	0.577	0.358	0.783
175	1	2.127	0.538	-1.589	0.581	0.359	0.786
176	1	2.55	0.486	-2.064	0.598	0.343	0.732
177	1	1.441	0.517	-0.923	0.57	0.368	0.818
178	1	2.145	0.616	-1.529	0.572	0.367	0.816
179	1	1.078	0.497	-0.581	0.563	0.374	0.838
180	1	2.118	0.51	-1.607	0.585	0.354	0.77
181	1	2.628	0.772	-1.856	0.571	0.37	0.826
182	1	1.322	0.561	-0.761	0.56	0.378	0.852
183	1	1.322	0.561	-0.761	0.56	0.378	0.852
184	1	1.078	0.497	-0.581	0.563	0.374	0.838
185	1	2.107	0.484	-1.623	0.591	0.349	0.752
186	1	1.441	0.517	-0.923	0.57	0.368	0.818
187	1	2.144	0.607	-1.537	0.573	0.367	0.813
188	1	1.438	0.505	-0.934	0.572	0.366	0.81
189	1	2.628	0.772	-1.856	0.571	0.37	0.826
190	1	2.099	0.465	-1.633	0.596	0.344	0.735
191	1	0.772	0.457	-0.315	0.557	0.377	0.85
192	1	1.248	0.711	-0.537	0.546	0.392	0.906

193	1	0.78	0.68	-0.101	0.526	0.41	0.974
194	1	1.441	0.517	-0.923	0.57	0.368	0.818
195	1	1.438	0.505	-0.934	0.572	0.366	0.81
196	1	2.145	0.616	-1.529	0.572	0.367	0.816
197	1	1.438	0.505	-0.934	0.572	0.366	0.81
198	1	1.246	0.461	-0.786	0.578	0.359	0.786
199	1	1.432	0.476	-0.955	0.578	0.359	0.787
200	1	2.94	15.426	12.485	0.057	0.906	13.583
201	1	1.262	0.558	-0.704	0.559	0.379	0.856
202	1	1.234	37.09	35.856	0.001	0.995	306.962
203	1	0.837	0.534	-0.303	0.545	0.391	0.901
204	1	1.438	0.505	-0.934	0.572	0.366	0.81
205	1	2.119	0.514	-1.605	0.585	0.355	0.773
206	1	0.772	0.456	-0.317	0.558	0.376	0.848
207	1	1.438	0.505	-0.934	0.572	0.366	0.81
208	1	2.119	0.514	-1.605	0.585	0.355	0.773
209	1	2.145	0.616	-1.529	0.572	0.367	0.816
210	1	1.248	0.711	-0.537	0.546	0.392	0.906
211	1	0.64	0.537	-0.102	0.525	0.409	0.973
212	1	2.149	8.576	6.426	0.117	0.84	7.359
213	1	1.48	0.617	-0.863	0.558	0.38	0.86
214	1	1.073	0.905	-0.168	0.531	0.407	0.963
215	1	2.184	37.736	35.552	0.005	0.982	74.843
216	1	13.352	5.029	-8.323	0.593	0.305	0.617
217	1	1.327	21.872	20.545	0.007	0.979	64.168
218	1	2.119	0.514	-1.605	0.585	0.355	0.773
219	1	2.118	0.51	-1.607	0.585	0.354	0.77
220	1	1.246	0.459	-0.787	0.579	0.358	0.784
221	1	1.441	0.517	-0.923	0.57	0.368	0.818
222	1	0.941	0.555	-0.386	0.547	0.389	0.894
223	1	2.107	0.484	-1.623	0.591	0.349	0.752
224	1	1.248	0.711	-0.537	0.546	0.392	0.906
225	1	1.248	0.711	-0.537	0.546	0.392	0.906
226	1	0.8	0.81	0.01	0.521	0.415	0.998
227	1	2.119	0.514	-1.605	0.585	0.355	0.773
228	1	2.119	0.514	-1.605	0.585	0.355	0.773
229	1	2.127	0.538	-1.589	0.581	0.359	0.786
230	1	2.118	0.51	-1.607	0.585	0.354	0.77
231	1	1.246	0.459	-0.787	0.579	0.358	0.784
232	1	1.438	0.505	-0.934	0.572	0.366	0.81
233	1	2.145	0.616	-1.529	0.572	0.367	0.816
234	1	0.949	0.783	-0.166	0.531	0.406	0.961
235	1	1.441	0.517	-0.923	0.57	0.368	0.818
236	1	2.145	0.616	-1.529	0.572	0.367	0.816
237	1	2.127	0.538	-1.589	0.581	0.359	0.786
238	1	2.118	0.51	-1.607	0.585	0.354	0.77
239	1	2.127	0.538	-1.589	0.581	0.359	0.786
240	1	1.441	0.517	-0.923	0.57	0.368	0.818
241	1	2.099	0.465	-1.633	0.596	0.344	0.735

242	1	1.246	0.461	-0.786	0.578	0.359	0.786
243	1	1.246	0.461	-0.786	0.578	0.359	0.786
244	1	1.246	0.461	-0.786	0.578	0.359	0.786
245	1	0.62	0.481	-0.138	0.532	0.401	0.939
246	1	1.262	0.558	-0.704	0.559	0.379	0.856
247	1	1.441	0.517	-0.923	0.57	0.368	0.818
248	1	1.047	0.438	-0.608	0.579	0.357	0.778
249	1	1.262	0.558	-0.704	0.559	0.379	0.856
250	1	1.248	0.711	-0.537	0.546	0.392	0.906
251	1	2.119	0.514	-1.605	0.585	0.355	0.773
252	1	1.253	0.493	-0.76	0.57	0.368	0.816
253	1	1.049	0.453	-0.596	0.574	0.362	0.797
254	1	1.049	0.452	-0.597	0.575	0.362	0.795
255	1	2.119	0.514	-1.605	0.585	0.355	0.773
256	1	0.933	0.459	-0.474	0.567	0.369	0.82
257	1	1.322	0.561	-0.761	0.56	0.378	0.852
258	1	0.837	0.534	-0.303	0.545	0.391	0.901
259	1	1.438	0.505	-0.934	0.572	0.366	0.81
260	1	0.837	0.534	-0.303	0.545	0.391	0.901
261	1	1.245	0.455	-0.79	0.58	0.357	0.779
262	1	2.095	7.069	4.974	0.133	0.821	6.462
263	1	2.099	0.465	-1.633	0.596	0.344	0.735
264	1	1.322	0.561	-0.761	0.56	0.378	0.852
265	1	2.118	0.51	-1.607	0.585	0.354	0.77
266	1	1.248	0.711	-0.537	0.546	0.392	0.906
267	1	2.118	0.51	-1.607	0.585	0.354	0.77
268	1	2.118	0.51	-1.607	0.585	0.354	0.77
269	1	2.144	0.607	-1.537	0.573	0.367	0.813
270	1	1.251	0.482	-0.769	0.572	0.365	0.807
271	1	1.441	0.517	-0.923	0.57	0.368	0.818
272	1	2.144	0.607	-1.537	0.573	0.367	0.813
273	1	0.936	0.495	-0.441	0.557	0.379	0.856
274	1	2.127	0.538	-1.589	0.581	0.359	0.786
275	1	2.118	0.51	-1.607	0.585	0.354	0.77
276	1	1.441	0.517	-0.923	0.57	0.368	0.818
277	1	2.119	0.514	-1.605	0.585	0.355	0.773
278	1	0.62	0.477	-0.143	0.533	0.399	0.934
279	1	1.262	0.558	-0.704	0.559	0.379	0.856
280	1	2.118	0.51	-1.607	0.585	0.354	0.77
281	1	0.933	0.459	-0.474	0.567	0.369	0.82
282	1	1.049	0.453	-0.596	0.574	0.362	0.797
283	1	0.933	0.459	-0.474	0.567	0.369	0.82
284	1	0.837	0.534	-0.303	0.545	0.391	0.901
285	1	0.777	0.553	-0.224	0.537	0.398	0.928
286	1	2.628	0.772	-1.856	0.571	0.37	0.826
287	1	1.438	0.505	-0.934	0.572	0.366	0.81
288	1	2.127	0.538	-1.589	0.581	0.359	0.786
289	1	1.441	0.517	-0.923	0.57	0.368	0.818
290	1	2.026	32.133	30.107	0.007	0.978	62.837

291	1	1.24	0.433	-0.807	0.588	0.348	0.751
292	1	2.145	0.616	-1.529	0.572	0.367	0.816
293	1	2.001	36.316	34.316	0.005	0.983	81.294
294	1	1.248	0.711	-0.537	0.546	0.392	0.906
295	1	0.62	0.477	-0.143	0.533	0.399	0.934
296	1	1.246	0.461	-0.786	0.578	0.359	0.786
297	1	1.441	0.517	-0.923	0.57	0.368	0.818
298	1	2.118	0.51	-1.607	0.585	0.354	0.77
299	1	0.941	0.555	-0.386	0.547	0.389	0.894
300	1	2.127	0.538	-1.589	0.581	0.359	0.786
301	1	0.941	0.555	-0.386	0.547	0.389	0.894
302	1	1.24	0.433	-0.807	0.588	0.348	0.751
303	1	0.77	0.43	-0.34	0.568	0.366	0.812
304	1	0.756	0.496	-0.259	0.545	0.389	0.895
305	1	0.756	0.496	-0.259	0.545	0.389	0.895
306	1	1.432	0.476	-0.955	0.578	0.359	0.787
307	1	1.438	0.505	-0.934	0.572	0.366	0.81
308	1	1.248	0.711	-0.537	0.546	0.392	0.906
309	1	2.127	0.538	-1.589	0.581	0.359	0.786
310	1	1.252	0.487	-0.765	0.571	0.366	0.811
311	1	0.936	0.495	-0.441	0.557	0.379	0.856
312	1	1.049	0.453	-0.596	0.574	0.362	0.797
313	1	1.322	0.561	-0.761	0.56	0.378	0.852
314	1	2.118	0.51	-1.607	0.585	0.354	0.77
315	1	1.078	0.497	-0.581	0.563	0.374	0.838
316	1	1.224	0.532	-0.692	0.561	0.376	0.846
317	1	1.064	0.606	-0.459	0.547	0.39	0.897
318	1	2.118	0.51	-1.607	0.585	0.354	0.77
319	1	1.441	0.517	-0.923	0.57	0.368	0.818
320	1	1.049	0.453	-0.596	0.574	0.362	0.797
321	1	0.753	0.511	-0.242	0.542	0.393	0.908
322	1	1.248	0.711	-0.537	0.546	0.392	0.906
323	1	2.55	0.486	-2.064	0.598	0.343	0.732
324	1	1.224	0.532	-0.692	0.561	0.376	0.846
325	1	1.441	0.517	-0.923	0.57	0.368	0.818
326	1	0.836	0.518	-0.318	0.547	0.388	0.891
327	1	2.099	0.465	-1.633	0.596	0.344	0.735
328	1	1.322	0.561	-0.761	0.56	0.378	0.852
329	1	1.252	0.487	-0.765	0.571	0.366	0.811
330	1	1.246	0.459	-0.787	0.579	0.358	0.784
331	1	1.322	0.561	-0.761	0.56	0.378	0.852
332	1	0.932	0.457	-0.476	0.568	0.368	0.818
333	1	1.224	0.532	-0.692	0.561	0.376	0.846
334	1	2.145	0.616	-1.529	0.572	0.367	0.816
335	1	1.438	0.505	-0.934	0.572	0.366	0.81
336	1	0.949	0.783	-0.166	0.531	0.406	0.961
337	1	1.078	0.497	-0.581	0.563	0.374	0.838
338	1	1.224	0.532	-0.692	0.561	0.376	0.846
339	1	0.8	0.81	0.01	0.521	0.415	0.998

340	1	1.441	0.517	-0.923	0.57	0.368	0.818
341	1	0.638	0.542	-0.096	0.524	0.41	0.977
342	1	1.441	0.517	-0.923	0.57	0.368	0.818
343	1	2.145	0.616	-1.529	0.572	0.367	0.816
344	1	0.837	0.534	-0.303	0.545	0.391	0.901
345	1	2.119	0.514	-1.605	0.585	0.355	0.773
346	1	1.441	0.517	-0.923	0.57	0.368	0.818
347	1	2.118	0.51	-1.607	0.585	0.354	0.77
348	1	2.118	0.51	-1.607	0.585	0.354	0.77
349	1	1.441	0.517	-0.923	0.57	0.368	0.818
350	1	1.438	0.505	-0.934	0.572	0.366	0.81
351	1	1.262	0.558	-0.704	0.559	0.379	0.856
352	1	2.127	0.538	-1.589	0.581	0.359	0.786
353	1	1.441	0.517	-0.923	0.57	0.368	0.818
354	1	0.64	0.537	-0.102	0.525	0.409	0.973
355	1	1.224	0.532	-0.692	0.561	0.376	0.846
356	1	2.119	0.514	-1.605	0.585	0.355	0.773
357	1	1.441	0.517	-0.923	0.57	0.368	0.818
358	1	1.246	0.461	-0.786	0.578	0.359	0.786
359	1	1.054	0.486	-0.568	0.565	0.372	0.831
360	1	1.246	0.459	-0.787	0.579	0.358	0.784
361	1	2.118	0.51	-1.607	0.585	0.354	0.77
362	1	1.224	0.532	-0.692	0.561	0.376	0.846
363	1	1.078	0.497	-0.581	0.563	0.374	0.838
364	1	2.118	0.51	-1.607	0.585	0.354	0.77
365	1	1.322	0.561	-0.761	0.56	0.378	0.852
366	1	1.224	0.532	-0.692	0.561	0.376	0.846
367	1	0.936	0.495	-0.441	0.557	0.379	0.856
368	1	1.253	0.493	-0.76	0.57	0.368	0.816
369	1	1.246	0.461	-0.786	0.578	0.359	0.786
370	1	2.118	0.51	-1.607	0.585	0.354	0.77
371	1	2.145	0.616	-1.529	0.572	0.367	0.816
372	1	2.628	0.772	-1.856	0.571	0.37	0.826
373	1	2.119	0.514	-1.605	0.585	0.355	0.773
374	1	2.118	0.51	-1.607	0.585	0.354	0.77
375	1	0.8	0.81	0.01	0.521	0.415	0.998
376	1	1.047	0.438	-0.608	0.579	0.357	0.778
377	1	2.119	0.514	-1.605	0.585	0.355	0.773
378	1	1.078	0.497	-0.581	0.563	0.374	0.838
379	1	1.441	0.517	-0.923	0.57	0.368	0.818
380	1	1.248	0.711	-0.537	0.546	0.392	0.906
381	1	0.941	0.555	-0.386	0.547	0.389	0.894
382	1	0.936	0.495	-0.441	0.557	0.379	0.856
383	1	1.441	0.517	-0.923	0.57	0.368	0.818
384	1	1.322	0.561	-0.761	0.56	0.378	0.852
385	1	1.049	0.453	-0.596	0.574	0.362	0.797
386	1	0.933	0.459	-0.474	0.567	0.369	0.82
387	1	2.119	0.514	-1.605	0.585	0.355	0.773
388	1	0.949	0.783	-0.166	0.531	0.406	0.961

389	1	3.383	25.313	21.929	0.034	0.934	19.905
390	1	2.145	0.616	-1.529	0.572	0.367	0.816
391	1	1.496	2.671	1.175	0.296	0.649	2.602
392	1	1.045	0.427	-0.617	0.584	0.352	0.762
393	1	0.756	0.496	-0.259	0.545	0.389	0.895
394	1	2.119	0.514	-1.605	0.585	0.355	0.773
395	1	1.441	0.517	-0.923	0.57	0.368	0.818
396	1	2.231	46.168	43.937	0.001	0.989	122.588
397	1	2.119	0.514	-1.605	0.585	0.355	0.773
398	1	2.118	0.51	-1.607	0.585	0.354	0.77
399	1	0.837	0.534	-0.303	0.545	0.391	0.901
400	1	1.252	0.487	-0.765	0.571	0.366	0.811
401	1	2.144	0.607	-1.537	0.573	0.367	0.813
402	1	1.245	0.455	-0.79	0.58	0.357	0.779
403	1	2.118	0.51	-1.607	0.585	0.354	0.77
404	1	1.038	24.677	23.639	0.002	0.991	155.91
405	1	2.628	0.772	-1.856	0.571	0.37	0.826
406	1	2.628	0.772	-1.856	0.571	0.37	0.826
407	1	1.441	0.517	-0.923	0.57	0.368	0.818
408	1	1.262	0.558	-0.704	0.559	0.379	0.856
409	1	1.322	0.561	-0.761	0.56	0.378	0.852
410	1	1.441	0.517	-0.923	0.57	0.368	0.818
411	1	1.441	0.517	-0.923	0.57	0.368	0.818
412	1	2.118	0.51	-1.607	0.585	0.354	0.77
413	1	0.941	0.555	-0.386	0.547	0.389	0.894
414	1	2.628	0.772	-1.856	0.571	0.37	0.826
415	1	2.119	0.514	-1.605	0.585	0.355	0.773
416	1	0.777	0.553	-0.224	0.537	0.398	0.928
417	1	1.224	0.532	-0.692	0.561	0.376	0.846
418	1	0.949	0.783	-0.166	0.531	0.406	0.961
419	1	2.628	0.772	-1.856	0.571	0.37	0.826
420	1	0.941	0.555	-0.386	0.547	0.389	0.894
421	1	1.054	0.486	-0.568	0.565	0.372	0.831
422	1	0.837	0.534	-0.303	0.545	0.391	0.901
423	1	2.118	0.51	-1.607	0.585	0.354	0.77
424	1	1.441	0.517	-0.923	0.57	0.368	0.818
425	1	1.246	0.461	-0.786	0.578	0.359	0.786
426	1	1.262	0.558	-0.704	0.559	0.379	0.856
427	1	2.127	0.538	-1.589	0.581	0.359	0.786
428	1	1.441	0.517	-0.923	0.57	0.368	0.818
429	1	1.438	0.505	-0.934	0.572	0.366	0.81
430	1	2.118	0.51	-1.607	0.585	0.354	0.77
431	1	2.118	0.51	-1.607	0.585	0.354	0.77
432	1	1.322	0.561	-0.761	0.56	0.378	0.852
433	1	1.438	0.505	-0.934	0.572	0.366	0.81
434	1	2.119	0.514	-1.605	0.585	0.355	0.773
435	1	1.224	0.532	-0.692	0.561	0.376	0.846
436	1	0.929	0.431	-0.498	0.577	0.358	0.783
437	1	1.432	0.476	-0.955	0.578	0.359	0.787

438	1	1.054	0.486	-0.568	0.565	0.372	0.831
439	1	1.054	0.486	-0.568	0.565	0.372	0.831
440	1	1.078	0.497	-0.581	0.563	0.374	0.838
441	1	2.628	0.772	-1.856	0.571	0.37	0.826
442	1	2.144	0.607	-1.537	0.573	0.367	0.813
443	1	2.107	0.484	-1.623	0.591	0.349	0.752
444	1	1.438	0.505	-0.934	0.572	0.366	0.81
445	1	2.628	0.772	-1.856	0.571	0.37	0.826
446	1	1.253	0.493	-0.76	0.57	0.368	0.816
447	1	2.118	0.51	-1.607	0.585	0.354	0.77
448	1	2.118	0.51	-1.607	0.585	0.354	0.77
449	1	2.145	0.616	-1.529	0.572	0.367	0.816
450	1	1.058	0.518	-0.54	0.558	0.379	0.855
451	1	0.834	0.484	-0.349	0.554	0.381	0.865
452	1	2.118	0.51	-1.607	0.585	0.354	0.77
453	1	1.441	0.517	-0.923	0.57	0.368	0.818
454	1	0.933	0.459	-0.474	0.567	0.369	0.82
455	1	1.441	0.517	-0.923	0.57	0.368	0.818
456	1	2.55	0.486	-2.064	0.598	0.343	0.732
457	1	1.224	0.532	-0.692	0.561	0.376	0.846
458	1	0.836	0.518	-0.318	0.547	0.388	0.891
459	1	1.246	0.461	-0.786	0.578	0.359	0.786
460	1	0.678	0.51	-0.168	0.534	0.4	0.934
461	1	0.837	0.534	-0.303	0.545	0.391	0.901
462	1	1.262	0.558	-0.704	0.559	0.379	0.856
463	1	1.438	0.505	-0.934	0.572	0.366	0.81
464	1	1.058	0.518	-0.54	0.558	0.379	0.855
465	1	1.441	0.517	-0.923	0.57	0.368	0.818
466	1	1.438	0.505	-0.934	0.572	0.366	0.81
467	1	1.441	0.517	-0.923	0.57	0.368	0.818
468	1	1.24	0.433	-0.807	0.588	0.348	0.751
469	1	2.127	0.538	-1.589	0.581	0.359	0.786
470	1	1.438	0.505	-0.934	0.572	0.366	0.81
471	1	2.118	0.51	-1.607	0.585	0.354	0.77
472	1	1.049	0.452	-0.597	0.575	0.362	0.795
473	1	2.119	0.514	-1.605	0.585	0.355	0.773
474	1	2.127	0.538	-1.589	0.581	0.359	0.786
475	1	1.441	0.517	-0.923	0.57	0.368	0.818
476	1	2.865	35.598	32.733	0.011	0.967	41.769
477	1	1.441	0.517	-0.923	0.57	0.368	0.818
478	1	2.127	0.538	-1.589	0.581	0.359	0.786
479	1	2.119	0.514	-1.605	0.585	0.355	0.773
480	1	0.949	0.783	-0.166	0.531	0.406	0.961
481	1	2.145	0.616	-1.529	0.572	0.367	0.816
482	1	1.441	0.517	-0.923	0.57	0.368	0.818
483	1	1.438	0.505	-0.934	0.572	0.366	0.81
484	1	2.628	0.772	-1.856	0.571	0.37	0.826
485	1	1.224	0.532	-0.692	0.561	0.376	0.846

Note

α	Mean posterior synonymous substitution rate at a site
β	Mean posterior nonsynonymous substitution rate at a site
β-α	Mean posterior nonsynonymous - synonymous substitution
Prob[α>β]	Posterior probability pf negative selection at a site
Prob[α<β]	Posterior probability pf positive selection at a site
BayesFactor[α<β]	Empiricial Bayes Factor for positive selection at a site
