# Supplementary material for: Genetic polymorphism and natural selection of the erythrocyte binding antigen 175 region II in Plasmodium falciparum populations from Myanmar and Vietnam
Source: Sci Rep. 2023 Nov 16;13:20025. doi: 10.1038/s41598-023-47275-6 (PMC10654615; doi:10.1038/s41598-023-47275-6)
Supplement: Supplementary file 3 — Supplementary Table S3. [file 41598_2023_47275_MOESM3_ESM.pdf]

Supplement File 3: Table S3. In/Del polymorphisms of the global *pfeba-175* RII

	H (i)	Hd (i)	π (i)
Vietnam	—	—	—
Myanmar	2	0.379	0.00026
Thailand	2	0.488	0.00034
Nigeria	2	0.458	0.00032
Kenya	2	0.146	0.00010
Benin	—	—	—
Madagascar	—	—	—
French Guiana	—	—	—
Colombia	—	—	—
Peru	—	—	—
Venezuela	—	—	—

H (i): Number of In/Del haplotypes; Hd (i): In/Del haplotype diversity;
Π (i): In/Del diversity per site.
