# Supplementary material for: Genetic polymorphism and natural selection of the erythrocyte binding antigen 175 region II in Plasmodium falciparum populations from Myanmar and Vietnam
Source: Sci Rep. 2023 Nov 16;13:20025. doi: 10.1038/s41598-023-47275-6 (PMC10654615; doi:10.1038/s41598-023-47275-6)
Supplement: Supplementary file 4 — Supplementary Table S4. [file 41598_2023_47275_MOESM4_ESM.pdf]

Supplement File 4: Table S4. Recombination events among the global *pfeba-175* RII populations

	Ra	Rb	Rm
Vietnam	0.0003	0.4	1
Myanmar	0.0273	39.7	6
Thailand	0.0158	22.9	4
Nigeria	0.0349	50.7	5
Kenya	0.020	29.1	6
Benin	0.0182	26.5	2
Madagascar	0.0455	66.2	3
French Guiana	0	0.001	2
Peru	0.0008	1.2	1
Colombia	0	0.001	0
Venezuela	0.0116	24.1	3

Ra: Recombination between adjacent sites; Rb: Recombination per gene;

Rm: Minimum number of recombination events
