# Supplementary figures and images for: Genetic polymorphism and natural selection of the erythrocyte binding antigen 175 region II in Plasmodium falciparum populations from Myanmar and Vietnam
Source: Sci Rep. 2023 Nov 16;13:20025. doi: 10.1038/s41598-023-47275-6 (PMC10654615; doi:10.1038/s41598-023-47275-6)

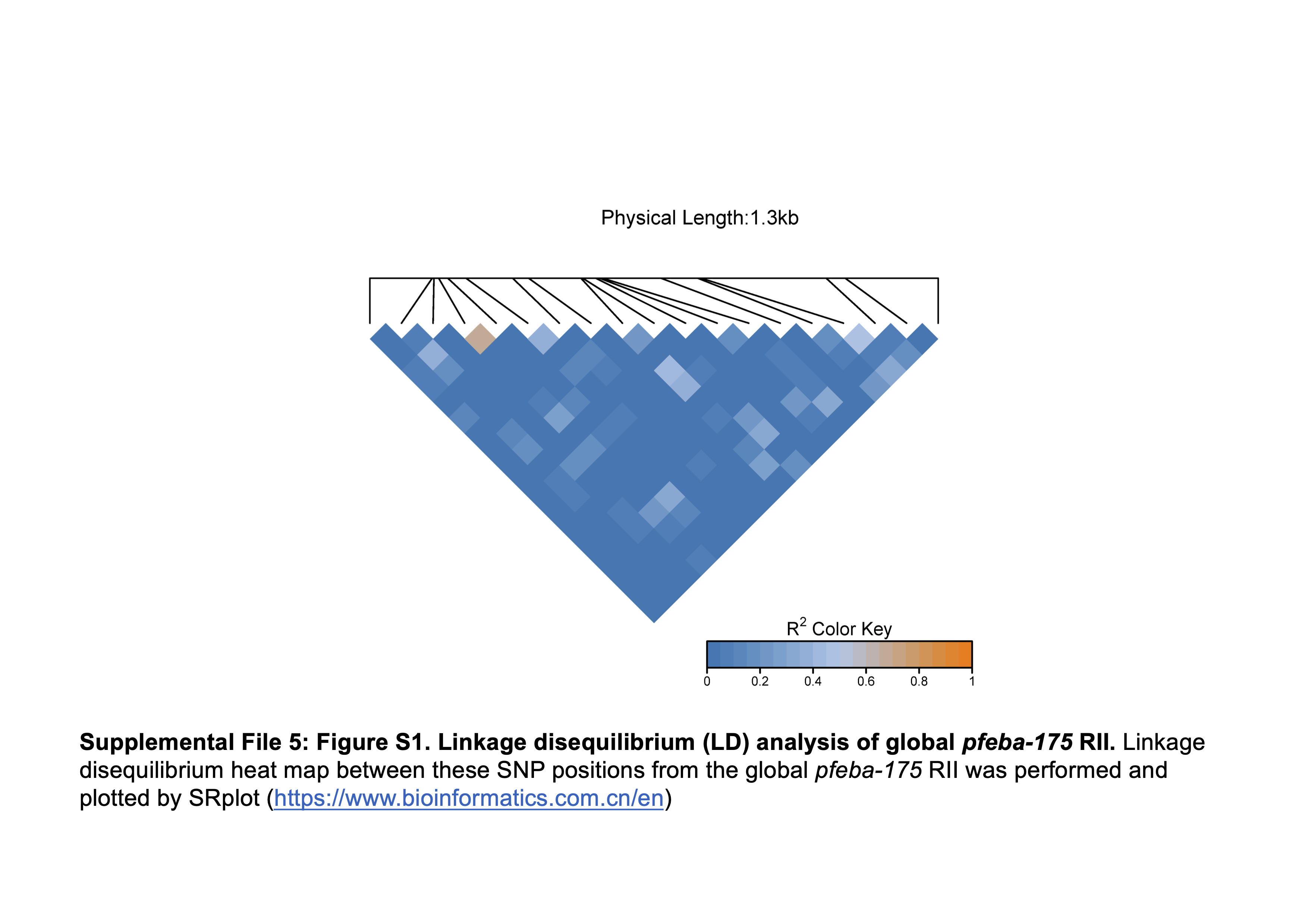

Supplement: Supplementary file 5 — Supplementary Figure S1. [file 41598_2023_47275_MOESM5_ESM.tif]

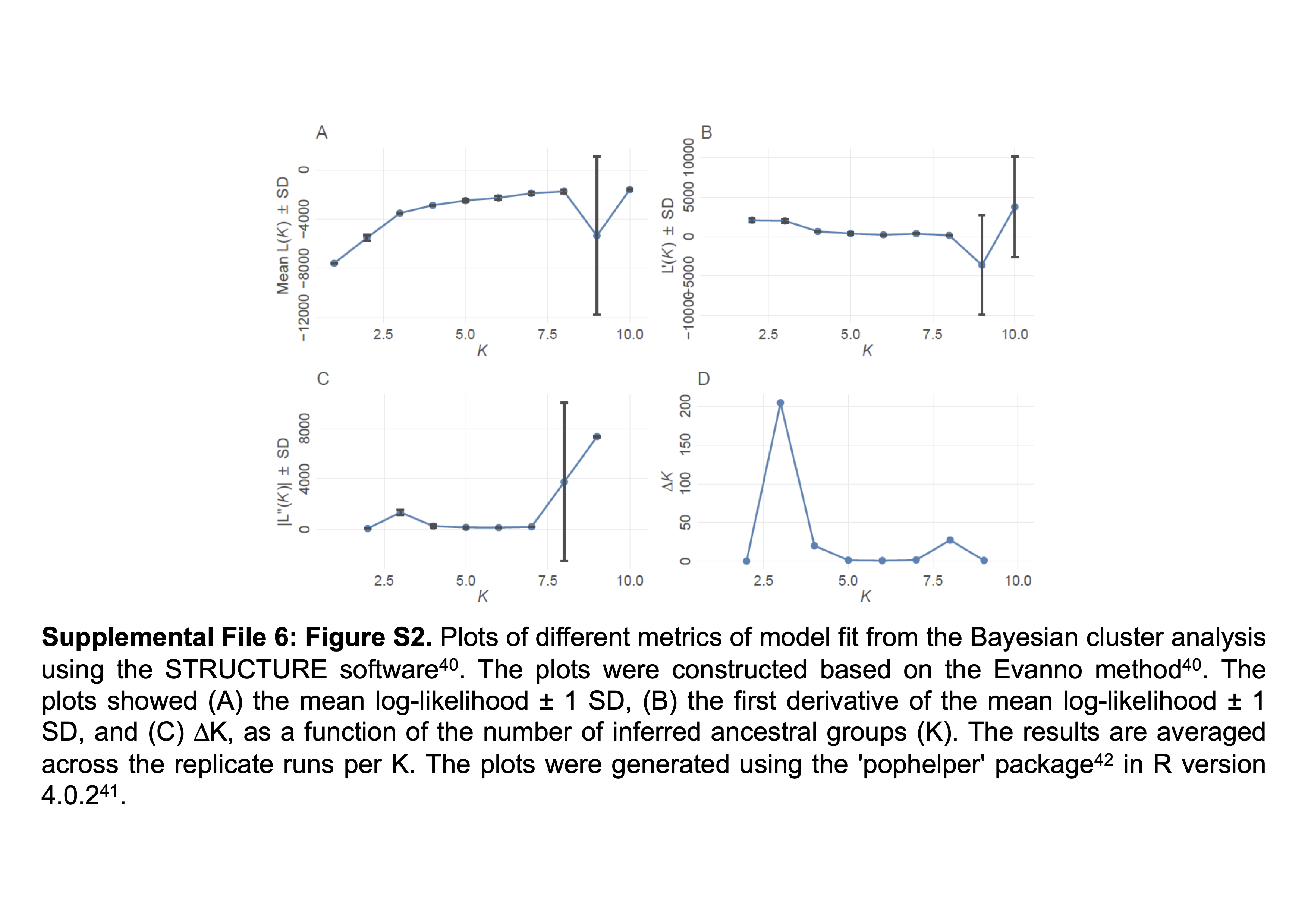

Supplement: Supplementary file 6 — Supplementary Figure S2. [file 41598_2023_47275_MOESM6_ESM.tif]
