# Supplementary material for: Genetic polymorphism and natural selection of the erythrocyte binding antigen 175 region II in Plasmodium falciparum populations from Myanmar and Vietnam
Source: Sci Rep. 2023 Nov 16;13:20025. doi: 10.1038/s41598-023-47275-6 (PMC10654615; doi:10.1038/s41598-023-47275-6)
Supplement: Supplementary file 7 — Supplementary Table S5. [file 41598_2023_47275_MOESM7_ESM.pdf]

Supplement File 7: Table S5. Global *pfeba-175* RII sequences analyzed in this study

Origin	Number of sequences	GenBank Accession No.	Reference
3D7	1	XP_001349207.2	
Benin	8	KJ419497-KJ419504	10.1111/mec.12696
Colombia	20	KJ419512-KJ419531	10.1111/mec.12696
French Guiana	62	KJ419532-KJ419546, KJ419586-KJ419632	10.1111/mec.12696
Kenya	39	DQ092087-DQ092125	10.1016/j.molbiopara.2006.05.010
Equatorial Guiana	49	MW691428–MW691476	10.1186/s12936-021-03904-x
Madagascar	7	KJ419505-KJ419511	10.1111/mec.12696
Nigeria	16	AJ438799, AJ438800, AJ438802, AJ438804, AJ438806, AJ438808, AJ438810- AJ438812, AJ438814, AJ438815, AJ438819, AJ438822-AJ438824, AJ438828	10.1093/genetics/163.4.1327
Peru	30	KJ419547-KJ419576	10.1111/mec.12696
Thailand	48	DQ092039-DQ092086	10.1016/j.molbiopara.2006.05.010
Venezuela	9	KJ419577-KJ419585	10.1111/mec.12696
