# Supplementary material for: Genetic polymorphism and natural selection of the erythrocyte binding antigen 175 region II in Plasmodium falciparum populations from Myanmar and Vietnam
Source: Sci Rep. 2023 Nov 16;13:20025. doi: 10.1038/s41598-023-47275-6 (PMC10654615; doi:10.1038/s41598-023-47275-6)
Supplement: Supplementary file 8 — Supplementary Legends. [file 41598_2023_47275_MOESM8_ESM.docx]

**Supplement Files Legends**

**Supplement File 1: Table S1.** Summary of nonsynonymous nucleotide polymorphisms

(SNPs) detected in Vietnam and Myanmar *pfeba-175* RII

**Supplement File 2: Table S2.** FUBAR test

**Supplement File 3: Table S3.** In/Del polymorphisms of the global *pfeba-175* RII

**Supplement File 4: Table S4.** Recombination events among the global *pfeba-175* RII populations

**Supplement File 5: Figure S1.** Linkage disequilibrium (LD) analysis of global *pfeba-175* RII.

**Supplement File 6: Figure S2.** Plots of different metrics of model fit from the Bayesian cluster analysis using the STRUCTURE software.

**Supplement File 7: Table S5.** Global *pfeba-175* RII sequences analyzed in this study
